# Reactivity and Mechanism of Recoverable Pd_1_@C_3_N_4_ Single-Atom Catalyst in Buchwald–Hartwig Aminations

**DOI:** 10.1021/acscatal.4c05134

**Published:** 2024-12-17

**Authors:** Georgios Giannakakis, Marc Eduard Usteri, Aram Bugaev, Andrea Ruiz-Ferrando, Dario Faust Akl, Núria López, Serena Fantasia, Kurt Püntener, Javier Pérez-Ramírez, Sharon Mitchell

**Affiliations:** † Institute of Chemical and Bioengineering, Department of Chemistry and Applied Biosciences, ETH Zurich, Vladimir-Prelog-Weg, 1, 8093 Zurich, Switzerland; ‡ Paul Scherrer Institute, Forschungsstrasse 111, 5232 Villigen, Switzerland; § Institute of Chemical Research of Catalonia (ICIQ), The Barcelona Institute of Science and Technology, Avenue Països Catalans 16, 43007 Tarragona, Spain; ⊥ Pharmaceutical Division, Synthetic Molecules Technical Development, Process Chemistry & Catalysis, F. Hoffmann-La Roche Ltd, 4070 Basel, Switzerland

**Keywords:** Buchwald−Hartwig amination, single-atom catalyst, palladium, reactivity trends, kinetics, surface-catalyzed mechanisms

## Abstract

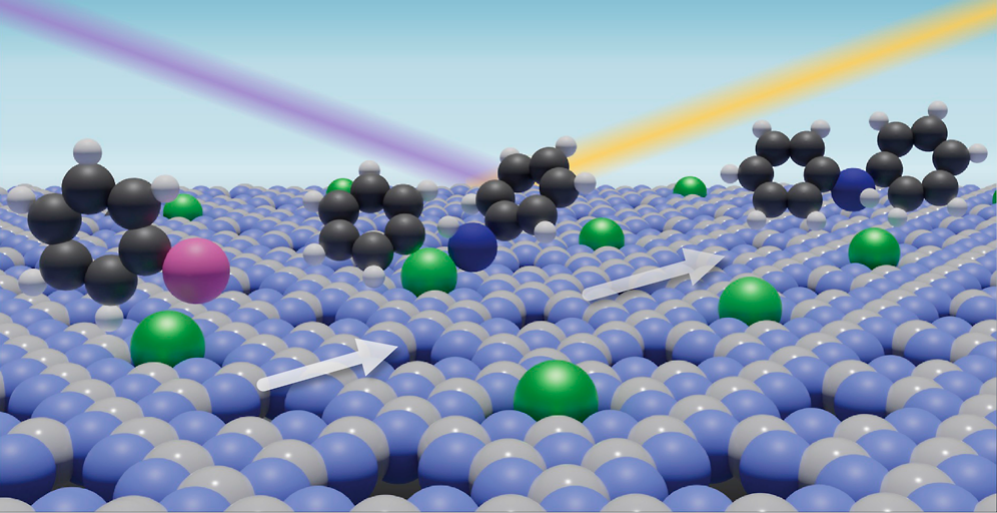

Buchwald–Hartwig (BH) aminations are crucial for synthesizing arylamine motifs in numerous bioactive molecules and fine chemicals. While homogeneous palladium complexes can be effective catalysts, their high costs and environmental impact motivate the search for alternative approaches. Heterogeneous palladium single-atom catalysts (SAC) offer promising recoverable alternatives in C–C cross-couplings. Yet their use in C–N couplings remains unexplored, and mechanistic insights into amine coupling with aryl halides over solid surfaces that could guide catalyst design are lacking. Here, we demonstrate that palladium atoms coordinated to well-defined heptazinic cavities of graphitic carbon nitride (Pd_1_@C_3_N_4_) deliver practically relevant yields for BH couplings across various aryl halides and amines, exhibiting persistent activity and negligible leaching over several cycles. Notably, Pd_1_@C_3_N_4_ shows comparable or superior activity with certain aryl chlorides to bromides, alongside high chemoselectivity for amines over amides. In situ X-ray absorption spectroscopy analyses supported by density functional theory simulations identify the concerted role of the ligand and the C_3_N_4_ host in determining the performance, with a Pd(II) nominal oxidation state observed under all coupling conditions. Complementary structural and kinetic studies highlight a distinct reaction mechanism than that typically reported for homogeneous catalysts. These findings offer key insights for designing recyclable SAC for BH coupling, setting the basis for extending the scope toward more complex industrial targets.

## 1 Introduction

Buchwald–Hartwig (BH) aminations have become a robust methodology for constructing arylamines in pharmaceutical, agro-, and fine chemical synthesis.^1−3^ The efficacy of these reactions stems from the use of palladium complexes as homogeneous catalysts, whose structures and operating environments have been highly optimized for coupling chemically diverse substrates.^4,5^ Recent computational studies, validated by experiments, have identified conditions and ligand–precatalyst combinations for guiding the synthesis of next-generation homogeneous catalysts.^6−10^ However, recovering these soluble catalytic systems remains difficult. A recent lifecycle analysis (LCA) underscored this as a crucial aspect to tackle in cross-coupling reactions due to the dominant environmental footprint of palladium, accounting for over 90% of all impacts calculated.^11^ This also poses a challenge for product purity, a crucial aspect in pharmaceutical compound synthesis, where palladium levels must often stay below 10 ppm.^12^

A direction of growing interest in addressing these issues is developing reusable heterogeneous palladium catalysts. Early studies focused on supported metal nanoparticles.^13−21^ However, challenges persist in controlling the palladium–support interaction to avoid metal loss via leaching^22,23^ or factors such as overcoordination of the active metal species limiting activity.^24,25^ Furthermore, mechanistic understanding of C–N bond formation via coupling amines with aryl halides on solid surfaces is lacking.^26^ Attaining the latter is hindered by the ill-defined nature of the systems reported and the evaluation of catalyst stability at high conversion. In this context, single-atom heterogeneous catalysts (SACs), offering more uniform and tailorable structures than supported metal nanoparticles,^27,28^ attract attention and have demonstrated high stability and selectivity in Suzuki–Miyaura (SM) and Sonogashira-Hagihara C–C couplings.^29−33^ LCA revealed that the possibility of recycling a stable heterogeneous catalyst even once could already bring environmental benefits compared to using palladium complexes even when considering up to 95% metal recovery for the homogeneous catalyst.^30^ Despite this initial promise, questions persist regarding the optimal design of SACs in C–C cross-couplings. For example, the adaptive coordination of metal atoms has been proposed as key to ensuring fulfillment of the catalytic cycle and high stability in Suzuki–Miyaura coupling but lacks direct evidence.^31^ Similarly, while Pd SACs based on functionalized carbons and carbon nitrides typically only perform well in the presence of ligands, the latter’s function is unclear.^30,31^ Although parallels have been suggested between SACs and homogeneous catalysts, the practical transferability of concepts between the two remains largely unexplored.^22^ Surprisingly, the reactivity of SACs in BH aminations remains unexplored despite comprising a key synthetic approach.

In this study, we demonstrate that palladium single atoms stabilized on a high surface area multidentate coordinating carbon nitride host (Pd_1_@C_3_N_4_), exhibit promising performance in BH aminations with negligible leaching under optimized conditions. The results are contextualized through benchmarking the yields and turnover frequencies (TOF) achieved with previously reported homogeneous and heterogeneous catalysts. From a synthetic perspective, Pd_1_@C_3_N_4_ displays promising flexibility to operate with distinct aryl halides and high chemoselectivity to amines over amides. Interestingly, depending on the electronic character of the substrate, modulated by the presence of electron-withdrawing or donating groups, we observed a nonclassical reactivity trend where aryl chlorides are activated as or more effectively than bromides or iodides, which also permits flexibility in terms of aryl halide use. The choice of a semicrystalline host material (C_3_N_4_) facilitates mechanistic insights via in situ characterization through X-ray absorption spectroscopy (XAS) coupled with complementary kinetic studies and simulations, shedding light on the surface-catalyzed reaction mechanisms. The results highlight distinct electronic and steric features of SACs compared to molecular catalysts, providing valuable insights for advancing their design.

## 2 Methods

### 2.1 Catalyst Synthesis

Pd_1_@C_3_N_4_ was synthesized following an established procedure.^25,31^ Dicyandiamide (20 g) was calcined at 550 °C (2.3 °C min^–1^ heating rate) in a ceramic crucible for 4 h under a nitrogen flow (15 mL min^–1^). The resulting powder was finely ground, and a portion (5 g) was thermally exfoliated at 500 °C (5 °C min^–1^ heating rate) for 5 h under static air, yielding a high surface area carbon nitride (C_3_N_4_) host. Palladium was introduced via microwave-irradiation-assisted deposition. The host material (2 g) was dispersed in deionized water (20 mL) by sonication for 1 h. Subsequently, an aqueous solution of ammonium tetrachloropalladate(II), (NH_4_)_2_PdCl_4_ (3.74 g_Pd_ L^–1^, 271.1 μL) was added dropwise and deionized water was added to reach a total volume of 50 mL. The resulting solution was stirred overnight. Microwave irradiation was carried out using a cyclic program of 15 s irradiation and 3 min cooling, repeated 20 times at 100 W power in a microwave reactor (CEM Discover SP). The solution was then vacuum filtered, washed with deionized water (500 mL, 3 cycles) and acetone (500 mL), and dried at 65 °C overnight. Finally, the sample was calcined at 300 °C for 5 h under N_2_ atmosphere.

### 2.2 Catalyst Characterization

The metal content in the catalyst was determined via inductively coupled plasma-optical emission spectroscopy (ICP-OES) using a Horiba Ultima 2 instrument (Kyoto, Japan). Before analysis, solids (typically, 10 mg) were digested overnight in a mixture of hydrogen peroxide (2 mL, 30 wt %, Thermo Fisher Scientific, Waltham, USA) and sulfuric acid (0.5 mL, 95 wt %, Sigma-Aldrich, Buchs, Switzerland). The digested samples were then diluted with MiliQ water to a total volume of 10 mL. Similarly, the palladium content remaining in the solution after using the catalyst in Buchwald–Hartwig (BH) amination or treatment with distinct solvent–ligand-base combinations was also determined via ICP-OES. After filtration of the catalyst, the reaction solution was dried at 65 °C overnight, and the sample preparation followed the same procedure as for solid samples. The metal dispersion in the catalyst was confirmed using high-angle annular dark-field scanning transmission electron microscopy mode (HAADF-STEM) imaging on a Talos F200X microscope (200 kV, Thermo Fisher Scientific, Waltham, U.S.), equipped with an energy dispersive X-ray spectroscopy (EDS) detector. Furthermore, the electronic and geometric structure of the catalyst were investigated by X-ray absorption spectroscopy (XAS), as described below.

### 2.3 XAS Investigation

For ex situ Pd *L*_3_-edge (*E*_0_ = 3.1733 keV) and P *K*-edge (*E*_0_ = 2.1455 keV) X-ray absorption near edge structure (XANES) measurements, we conducted experiments in vacuum at the PHOENIX I beamline of the Swiss Light Source at the Paul Scherrer Institute (PSI) in Villigen, Switzerland. A solid-state fluorescence detector (Ketek GmbH, Germany), positioned at a 90° angle with respect to the incoming beam, was used to measure the total fluorescence yield. Samples were prepared by drop-casting aliquots of the reaction solutions onto tape pieces fixed to a copper holder at predefined positions. The samples were then dried under N_2_ flow and inserted into the measurement chamber for XAS spectroscopy in fluorescence mode, typically acquiring 2–3 scans per sample.

In situ Pd *K*-edge (*E*_0_ = 24.3503 keV) XANES and extended X-ray absorption fine structure (EXAFS) measurements were conducted at the SuperXAS beamline of the Swiss Light Source.^34^ The incident photon beam provided by a 2.9 T superbend magnet was selected by a Si(111) channel-cut Quick-EXAFS monochromator oscillating at 1 Hz.^34^ The reaction vials were prepared as described in Section 2.4 and placed on the stirred heating stage. They were removed from the heating stage after 2 h and mounted on the motorized stage without stirring. The stir bar was not removed to minimize transfer time and prevent changes to the reaction atmosphere. Although the measurement of solid suspensions can be challenging due to inhomogeneous distributions of the catalyst particles, the suspension was sufficiently stable for measurements over 15–20 min. Herein, Quick-EXAFS enables the elimination of artifacts arising from inhomogeneities by measuring hundreds of spectra per sample, over which these effects average out. The vials were aligned with a focused X-ray beam (ca. 100μm diameter) to maximize the optical pathway through the sample. The signal in transmission mode was collected by Ar/N_2_ filled (ca. 10% absorption) ionization chambers. Palladium foil was measured simultaneously as the Pd(0) reference. For a sufficient signal-to-noise ratio, a typical spectrum was obtained by continuous collection and subsequent averaging over 15–20 min. To assess the potential impact of temporal changes in the spectra, Figure S1 provides an example in which the spectra collected during a single measurement were divided into two-halves and averaged separately. This approach confirmed the limited changes occurring during measurement, as the differences between the averaged spectra from the first and second halves were negligible. Raw data were processed using the ProQEXAFS software package,^35^ followed by data analysis using the Demeter package (see Note S1 for more details).^36^ The XANES descriptors presented in Table S1 were extracted using a parabolic fit and a Gaussian fit of the first feature in the *K*-edge spectra and their derivative spectra, respectively (Figure S2).

### 2.4 Catalyst Evaluation

Standard batch catalytic tests were conducted in sealed vials (total vial volume 2 mL). Typically, the reaction mixture contained Pd (0.01 equiv), ligand (0.1 equiv, PPh_3_ (triphenylphosphine), PCy_3_ (tricyclohexylphosphine), P*^t^*Bu_3_·HBF_4_ (tri-*tert*-butylphosphonium), or RuPhos (2-dicyclohexylphosphino-2′,6′-diisopropoxybiphenyl)), base (3 equiv, LiHMDS (lithium bis(trimethylsilyl)amide) or NaO^*t*^Bu (sodium *tert*-butoxide)), aryl halide (0.25 mmol, 0.25 M, 1 equiv), amine (1.5 equiv), and solvent (1 mL, toluene or anisole). As the bases were provided in tetrahydrofuran (THF), THF always formed part of the final solvent mixture in amounts corresponding to the base (50% for LiHMDS and 25% for NaO^*t*^Bu). The vials were agitated using magnetic stirrers at 900 rpm, and the temperature was maintained at 115 °C for 20 h. After reaction, the contents of the vials were centrifuged to separate the catalyst from the reaction mixture, with any remaining catalyst material further filtered out using disposable syringe filters (PTFE 45/25 Chromafil, Düren, Germany). Gas chromatography (GC) was performed using a ZB-5 column (5%-phenyl-95%dimethylpolysiloxane, 30 m length, 0.25 mm inner diameter, 0.25 mm film thickness) with He as the carrier gas, operated by a Thermo TRACE 1300 chromatograph equipped with a flame ionization detector. For analysis, the reaction solution (50 μL) was dissolved in acetonitrile (1 mL) and *n*-decane was added as an internal standard. The structural integrity of the ligand in the solution was assessed via ^31^P NMR spectroscopy using a 300 MHz Bruker spectrometer. Aliquots of the reaction solution were mixed in an NMR tube with *d*_3_-acetonitrile (CD_3_CN), and 128 scans of the ^31^P spectrum were acquired using the comp pulse decoupling (PCPD) method.

### 2.5 High-Throughput Catalyst Evaluation

The profiling of Pd_1_@C_3_N_4_ catalysts was performed at F. Hoffmann-La Roche’s facilities for automated high-throughput experimentation employing 96-well plate formats. All reactions were performed following a standardized BH testing protocol. As an example, the catalyst Pd_1_ @C_3_N_4_ (1 mol %, 0.01 equiv Pd), the ligand (0.1 equiv RuPhos) and the base (3 equiv NaO^*t*^Bu) were automatically dosed into a 1 mL reaction vial, followed by the halide (1 equiv, iodobenzene, 1.8 mg, 8.82 mmol, 0.19 M), the amine (1.5 equiv, morpholine) and toluene (45.90 μL) as the solvent. Finally, all individually configured 96 reaction vials were placed in a customized aluminum high-throughput testing unit and sealed airtight under a dry nitrogen atmosphere. The testing array was positioned on a heating stirrer in the fume hood, and the reaction temperature was set to 115 °C. Small magnetic stir bars were used to stir the reaction mixtures at 400 rpm for 4 h or 20 h before allowing them to gradually cool to room temperature. After opening the testing array at ambient conditions, the plate was analyzed by LC–MS. Selected reactions were analyzed by quantitative NMR on a 400 MHz Bruker spectrometer (Note S2) and the spectra are shown in the Supporting Information.

### 2.6 DFT Simulations

Density functional theory (DFT) simulations on slab models representing the different Pd_1_@C_3_N_4_ and derived systems were conducted using the Vienna ab initio simulation package (VASP 5.4.4).^37,38^ The generalized gradient approximation with the Perdew–Burke–Ernzerhof (GGA-PBE)^39^ functional was used to calculate the exchange–correlation energies, with dispersion contributions included,^40^ and spin-polarization was allowed. Core electrons were described by projector augmented waves (PAW),^41^ while valence monoelectronic states were expanded in plane waves with a cutoff energy of 450 eV. For single-species adsorption simulations, the Brillouin zone was sampled with a gamma-centered 3 × 3 × 1 *k* point grid and C_3_N_4_ was simulated as a heptazinic 2 × 2 supercell of four layers with the bottom one fixed to the bulk configuration. For coadsorption events, the Brillouin zone was sampled with a gamma-centered 2 × 2 × 1 *k* point grid and C_3_N_4_ was simulated as a heptazinic 4 × 4 monolayer supercell. Pd_1_@C_3_N_4_ was constructed by placing a Pd atom in the heptazinic cavity of the top and second layers. The structures of all investigated systems were relaxed using convergence criteria of 10^–4^ and 10^–5^ eV for the ionic and electronic steps, respectively. The arising dipole was corrected in all slab models.^42^ All structures presented in this work are available from the ioChem-BD database.^43,44^

## 3 Results and Discussion

### 3.1 SAC Optimization for BH Aminations

**Figure 1:**
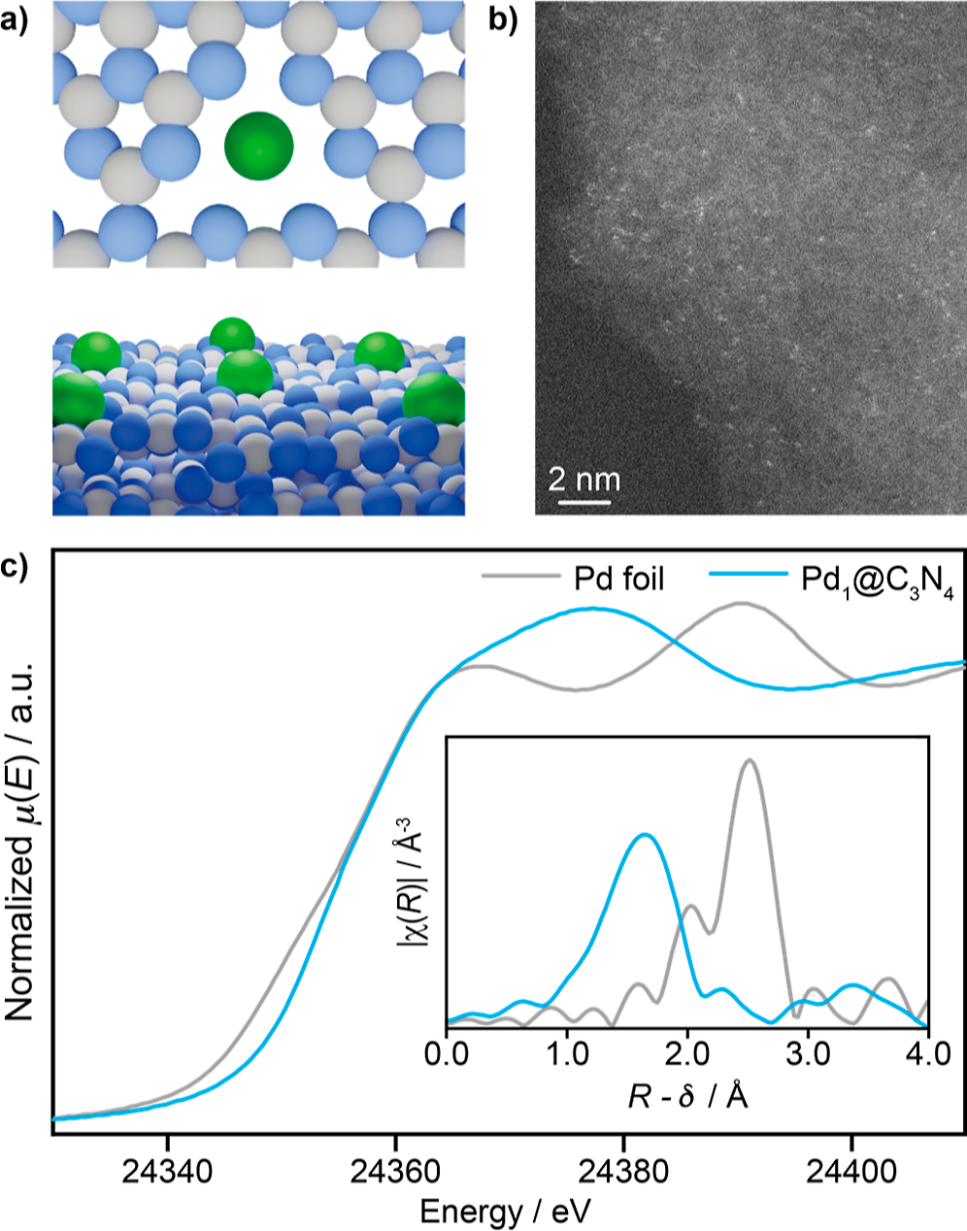
(a) Structural depiction (top and side views), (b) HAADF-STEM image and (c) ex situ Pd *K*-edge XANES spectra of the synthesized Pd_1_@C_3_N_4_ catalyst, compared to a metallic Pd(0) foil reference. The inset in (c) shows the Fourier-transformed EXAFS data.

To explore the potential of SACs in BH amination, we stabilized isolated palladium atoms on a high surface area form of carbon nitride (C_3_N_4_) known to contain abundant pyridinic nitrogen anchoring sites.^31^ Although other functionalized carbons can also be used as hosts for SACs,^30,32^ metal coordination sites in graphitic C_3_N_4_ are expected to be more well-defined (Figure 1a).^31^ The Pd_1_@C_3_N_4_ catalyst was prepared via a synthetic approach that is not air-sensitive with a palladium content of 0.5 wt % to ensure the presence of isolated atoms (see Methods). Scanning transmission electron microscopy (STEM) imaging confirmed the presence of isolated palladium atoms in the resulting material (Figure 1b) and X-ray absorption near edge structure (XANES) and extended X-ray absorption fine structure (EXAFS) analyses demonstrated the absence of Pd–Pd contributions and a typical total coordination to the support that corresponds to 3 neighboring atoms (Pd–C/N/O) (Figure 1c).

Choosing morpholine and bromobenzene as representative substrates, the catalytic activity in BH coupling was initially studied under a broad range of conditions for 20 h (Table S2), exploring the choice of solvent (toluene, anisole, or 1,2-dimethoxyethane/water mixture), base (LiHMDS, NaO^*t*^Bu, or K_2_CO_3_), and various ligands (RuPhos, PPh_3_, PCy_3_, or P^*t*^Bu_3_·HBF_4_), and their corresponding amount (5 or 10 mol %). All reagents were chosen because of their suitability and common use for activating the substrates in practically applied homogeneously catalyzed processes.^20,45−48^ Appreciable yields were demonstrated at elevated temperatures (115 °C) for most water-free cases. However, no reactivity was observed if water was present in the solvent mixture. For strong bases like LiHMDS and NaO^*t*^Bu, this result is consistent with the fact that even traces of water can convert them into a relatively weak hydroxide.^45,49^ Nevertheless, it is known that hydroxide and carbonate bases may be employed in BH aminations using polar solvents such as DME depending on the coupling partners and catalyst.^50^ This was not the case for Pd_1_@C_3_N_4_, which required aprotic conditions and strong bases under this initial set of conditions.

All the ligands studied could promote appreciable yields depending on the conditions (Table S2). While it was difficult to pinpoint general trends, the base–ligand combination had a major impact. With NaO^*t*^Bu, PCy_3_ shows negligible conversion using 5 or 10 mol %, while RuPhos and P^*t*^Bu_3_·HBF_4_ show higher yields that increase with ligand amount at short reaction times (*t* = 2 h). Interestingly, PPh_3_ gave moderate yields despite normally being mostly unreactive for this reaction. Furthermore, an inverse trend was observed with PPh_3_ amount with 5 mol % associated with improved yields. The moderated impact of PPh_3_ may be caused by its susceptibility to oxidation^51^ as using the oxidized form OPPh_3_ led to negligible yields, like those obtained under ligand-free conditions (Figure S3). The oxidation of PPh_3_ was confirmed experimentally (^31^P NMR in Figure S4) but was not observed for Ruphos. If LiHMDS is used instead, these trends mostly disappear as the different ligands give more similar yields. While LiHMDS normally works well in applications involving substrates bearing acidic or protic functionalities^50^ it was included in this study as it was hypothesized to interact favorably with surface nitrogen atoms in C_3_N_4_ in addition to successful implementation in a previous study.^52^ This could, in turn, explain the observed leveling of performances among the ligands. A critical aspect of heterogeneous catalyst design concerns maintaining the structural integrity of the active surface sites during use. Analysis of the palladium content (Table S3) and dispersion (Figure S5) in the materials recovered after catalytic evaluation under selected conditions confirmed the robustness of Pd_1_@C_3_N_4_. Interestingly, tests involving PPh_3_ and P^*t*^Bu_3_·HBF_4_ led to nanoparticle formation (Figure S5). This behavior could result from leaching and redeposition of metal species or surface migration. The higher Pd contents observed in the solution phase after catalyst removal suggest that leaching occurs to some extent under these conditions. For this reason, PPh_3_ and P^*t*^Bu_3_·HBF_4_ were excluded as potential ligands. We note that previous investigations of palladium nanoparticle-based catalysts in C–C cross-coupling reactions have linked palladium leaching to increased catalyst activity.^22^ In our case, under the conditions that promote nanoparticle formation potentially via leaching (Entry 7, Table S3), limited activity was observed (Table S2). This observation also agrees with the inability of PPh_3_ to work in homogeneous catalysts. Following this two-pronged reactivity and structural stability assessment, general conditions are identified for further exploration using RuPhos (at 10 mol %), toluene as the solvent, and either LiHMDS or NaO^*t*^Bu as the base.

### 3.2 Substrate Scope and Catalyst Stability

The preferential activation of specific functional and leaving groups in BH aminations is heavily influenced by the ligands of homogeneous catalysts, indicating the importance of the geometric and electronic properties of the active site.^29^^,^^52^ Therefore, it is interesting to investigate the knowledge transferability from homogeneous to heterogeneous systems, particularly in understanding the potential scope and reactivity patterns over the Pd_1_@C_3_N_4_ catalyst.

**Figure 2:**
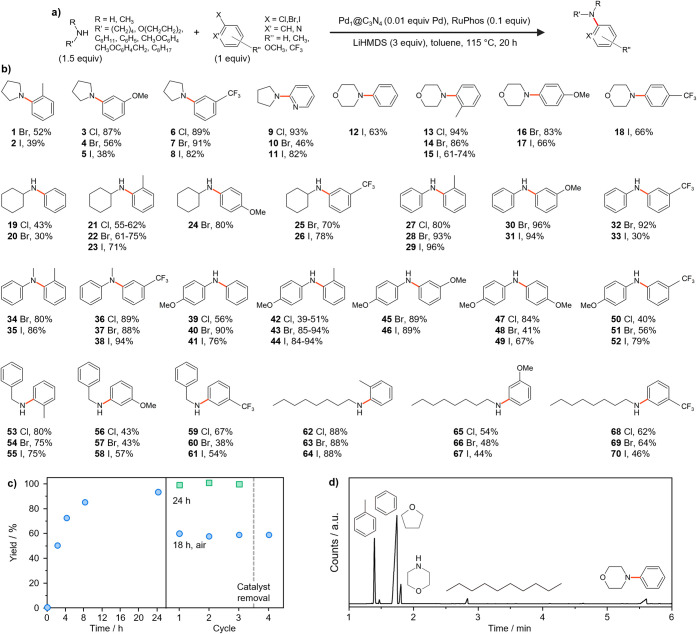
(a) Standardized protocol for high-throughput evaluation of the Pd_1_@C_3_N_4_ catalyst in BH aminations and substrate scope. (b) Products obtained with yields exceeding 30% and negligible background contributions using LiHMDS base. The full data set is presented in Tables S3 and S4. Yields refer to percentage area of product determined by LC–MS. (c) Kinetic and stability evaluation of Pd_1_@C_3_N_4_ in the BH coupling of morpholine and bromobenzene in batch tests under the same conditions apart from the time (indicated) and operating in air. Cycle 4 shows the yield observed 20 h after catalyst removal by hot filtration. (d) Typical gas chromatogram produced by the reaction mixtures obtained in (c). Peak assignments are shown inset.

Utilizing LiHMDS and RuPhos as a more promising base and ligand combination in terms of yield and stability, the coupling of a broad range of amines and amides was examined in combination with aryl halides bearing various substituents in the ortho-, meta , and para-positions on the benzene ring (halo(trifluoromethyl)benzenes, halotoluenes, halopyridines, haloanisoles) (Figure 2a,b, Tables S3 and S4) following a standardized high-throughput protocol (see Methods, Figure S6). Substrates were selected to maximize the variance in steric and electronic properties. Moderate to high yields were achieved for both primary and secondary amines and anilines coupled with all aryl halides, while negligible reactivity was observed for amides. This sensitivity to the amine type highlights the potential of Pd_1_@C_3_N_4_ SACs for chemoselective couplings.

Kinetic investigations involving morpholine and bromobenzene conversion to phenylmorpholine (Figure 2c) validate the heterogeneous character of the palladium catalyst, which remains immobilized and is not leached into soluble species. The yield evolution (Figure 2c) combined with the results presented in Table S2 suggests that reduction of ligand concentration would still allow reaching good yields over 20 h following further optimization, as 70% yield was exceeded after 4 h with the model substrates (morpholine and bromobenzene) and comparable yields were obtained for various other substrate combinations (Table S4 and S5). This is an important consideration considering the cost of ligands such as RuPhos. Moreover, operation outside an air-free environment obtains appreciable yields after 18 h (Figure 2c), demonstrating the catalysts’ robustness for scaled-up applications. Importantly, no further reactivity was observed in the solution obtained by removing the catalyst by filtration after cycle 3 even after treating it under reaction conditions for an additional 20 h (cycle 4).

The observed formation of minor amounts of benzene (Figure 2d) likely results from bromobenzene dehalogenation, a side reaction only observed when using LiHMDS. This undesired reactivity highlights the importance of considering the potential effects of specific reagent combinations (catalyst-base-ligand) on performance. Blank tests in the absence of the Pd_1_@C_3_N_4_ catalyst but in the presence of LiHMDS and ligand revealed background reactivity for various C–N coupling combinations (Tables S4 and S5), which is in line with previous studies.^53^ Additionally, the decreased yields of some products over the reaction course (higher yields after 4 h than 20 h, Tables S4 and S5) suggest potential product decomposition over time. The product decomposition could be mediated by LiHMDS as the base, as a similar behavior was also observed in the absence of the catalyst.

### 3.3 Leaving Group Activation Preference

**Figure 3:**
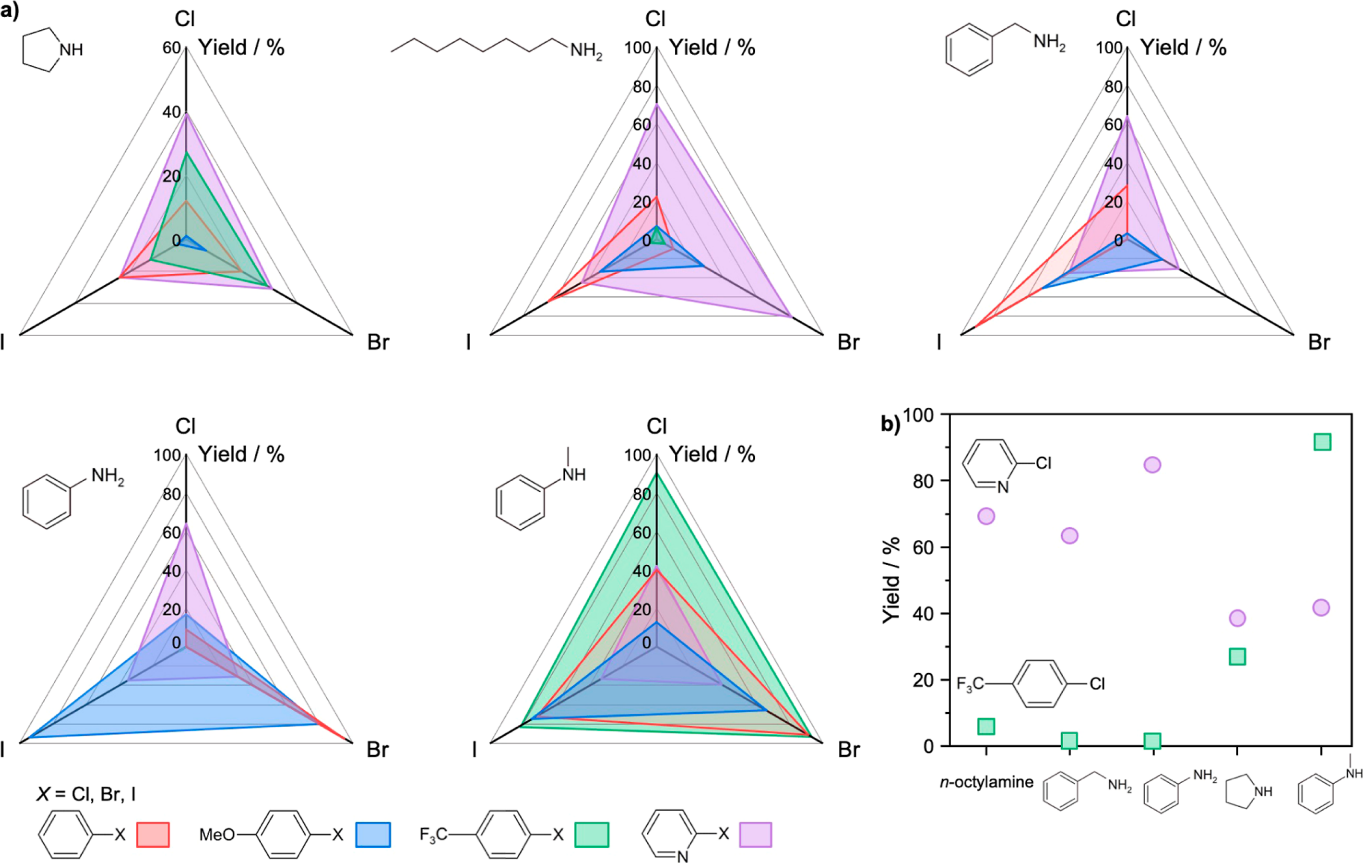
(a) Performance of the Pd_1_@C_3_N_4_ catalyst in the BH coupling of distinct amines and aryl halides. (b) Trends in the BH amination of primary and secondary amines with electron-withdrawing aryl halides, 2-chloropyridine and 4-chloro(trifluoro)benzene. Reaction conditions follow the standardized protocol for high-throughput evaluation using NaO^*t*^Bu as base. Yields refer to the percentage area of the product determined by LC–MS.

Since no background activity was observed when using NaO^*t*^Bu, further exploration of the reaction scope was performed using this base (Table S6). Like the tests using LiHMDS, moderate to high yields are observed for various coupling pairs of amines and aryl halides (Figure 3), yet no reactivity was measured in coupling of aryl halides with pyrrole, benzamide, or benzenesulfonamide (Table S6). In palladium-catalyzed coupling reactions, the reactivity trend for both homogeneous and heterogeneous catalysts is typically reported to follow the carbon–halogen bond strength. It is noteworthy that in some previous reports on heterogeneous catalysts, iodo compounds coupled more efficiently than aryl bromides or chlorides, with the latter considered as least active.^14,18,20^ Similarly, the Pd_1_@C_3_N_4_ catalyst also shows this trend in the couplings of halobenzenes and haloanisoles, with iodo compounds displaying greater reactivity than bromo or chloro analogs (Figure 3a, red and blue triangles).

Conversely, a notable deviation from this trend is observed in the case of halopyridines and 4-halo(trifluoromethyl)benzenes (Figure 3a, green and purple triangles), which are more electron-deficient aryls compared to the previous two. In these instances, the trend appears disrupted, as aryl chlorides are converted and coupled to the amines as efficiently or better than bromides and especially iodides (Table S7). Similar behavior is also evidenced when LiHMDS is used as the base for 3-halo(trifluoromethyl)benzene coupling with *N*-methylaniline, as well as for 2-halotoluene coupling with cyclohexylamine (Figure S7). Differences in electronic structure between Pd_1_@C_3_N_4_ and Pd-phosphine systems have previously been shown by XAS.^33^ Additionally, the steric effects imparted by the support surface likely influence catalyst performance. Accordingly, the inverse reactivity trend of some aryl halides might originate from steric or electronic interactions with the Pd_1_@C_3_N_4_ catalyst that differ from the commonly active ones in homogeneously catalyzed BH couplings.

In the context of electron-poor substrates, such as halopyridines and halo(trifluoromethyl)benzenes, another interesting behavior is noticeable (Figure 3b). Halopyridines exhibit significantly higher coupling yields with primary amines (70%, 64%, and 85% for octylamine, benzylamine, and aniline, respectively) compared to secondary amines (39% and 42% for pyrrolidine and *N*-methylamine, respectively). Conversely, halo(trifluoromethyl)benzenes show virtually no coupling with primary amines (6%, 0%, and 0% for octylamine, benzylamine, and aniline), but achieve higher yields with secondary amines, such as pyrrolidine and especially *N*-methylamine (27% and 92% yield, respectively). These results further underscore the crucial electronic and steric interaction between the catalytic surface and the reactants, leading to nontrivial reaction outcomes.

The preference of Pd_1_@C_3_N_4_ to couple C–Cl bonds as efficiently as C–Br or C–I for specific aryl halides is intriguing, diverging from classical homogeneous catalysis trends in coupling reactions. This trend normally arises when oxidative addition (OA) is rate-limiting. Deviation from it may imply changes in the rate-limiting step toward transmetalation in SM or amination in BH, as previously observed.^54^ Notably, this change to rate-limiting transmetalation was recently shown experimentally for SM reactions over Pd_1_@C_3_N_4_.^33^ As the electronic and steric demands for these steps are different from OA, trend inversions can occur. Alternatively, the deviation may also arise from intrinsic changes in C–Cl activation. The facile activation of C–Cl bonds is highly desirable from a practical standpoint as the optimal choice of halide for sustainable couplings may vary (vide infra) and improving the flexibility for working with distinct aryl halides has been the focus of some research groups. Selective coupling of (hetero)aryl C–Cl bonds over C–I or C–Br bonds was reported but is limited to substrates with activated C–Cl bonds.^55^ Similar reactivity of C–Cl and C–Br bond was reported under near to solvent-free conditions in SM cross-coupling.^56^ Finally, the preferential activation of activated chlorides over nonactivated ones has also been reported for heterogeneous catalysts, but no trend inversion among the halides is observed.^57^ Overall, this behavior has been linked to an electronic effect imparted by the active sites, as claimed in the case of bromo-compounds being more active than iodo substituents for the SM reaction.^58^ This argument could be further supported by the preferential activation of chloro substituents compared to iodo ones, for NiI_2_ and NiBr_2_ catalyzed SM cross coupling, although the classical trend was observed for BH coupling.^59^ Therefore, it could be hypothesized that the palladium sites assume a more Ni-like character in Pd_1_@C_3_N_4_, i.e., a change in their electronic density. Additionally, the higher steric requirements of iodide compared to chloride^60,61^ may be problematic due to the carrier surface imposing greater steric constraints on one side of the Pd site. Lastly, the effect of the solubility of the salt formed after aryl halide activation should not be overlooked, as has been shown for NaI in different solvents in amination reactions.^46,62^ Nonetheless, all three effects would individually lead to either trend inversion or retention for most substrates. Accordingly, a balance between multiple effects is likely the cause for trend inversion between different substrate classes.

Comparison of the turnover frequency (TOF) achieved with previously reported values for heterogeneous (Table S8) and homogeneous (Table S9) catalysts explored in the BH reaction confirms the potential, revealing similar values. While tailored homogeneous catalysts still exhibit substantially higher TOFs, our Pd_1_@C_3_N_4_ catalyst achieved values comparable to first-generation metal complexes (Table S9), indicating the potential for further optimization. Nevertheless, the practically relevant yields and stable performance obtained with the Pd_1_@C_3_N_4_ are promising from the point of view of reducing the environmental footprint of BH aminations by improving metal recovery. Furthermore, the uncommon reactivity observed warrants further investigation, which prompted us to conduct in situ studies to obtain more insights into the structural dynamics of the active sites and reaction mechanisms.

### 3.4 Electronic and Chemical State of Active Sites

Further advancing the design of Pd-SACs depends on the ability to control the interaction of palladium with the anchoring sites in the host. Despite recent advances in developing selective SACs for organic synthesis, there is limited understanding of the active site structure under reaction conditions.^22^ Interactions with the substrates, solvents, or other reagents in the reaction medium may change the local coordination environment of metal centers, impacting their reactivity and stability, especially at elevated temperatures. Therefore, assessing the effect of these variables on the active site architecture via in situ approaches, such as XAS, is crucial.^24,63^

**Figure 4:**
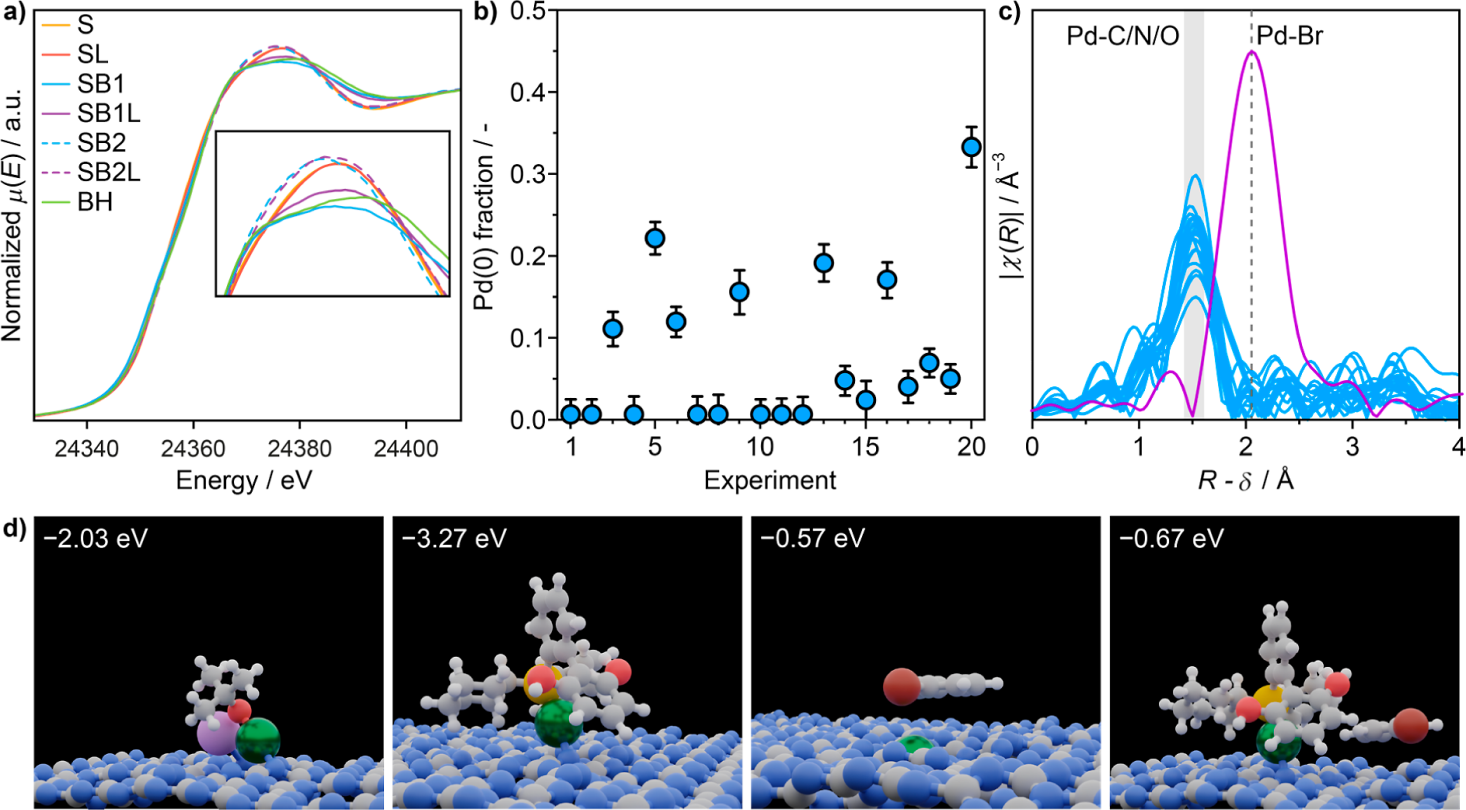
(a) Selected Pd *K*-edge XANES spectra of Pd_1_@C_3_N_4_ in distinct solvent (S), LiHMDS (B1), NaO^*t*^Bu (B2), ligand (L) mixtures and in the BH amination reaction. Reaction conditions follow the standardized protocol shown in Figure 2, using RuPhos as a ligand, LiHMDS or NaO^*t*^Bu as the base, and morpholine and bromobenzene as reagents in the reaction. (b) Fraction of Pd(0) from linear fitting combination analysis for all XAS measurements in the same order as described in Tables S1 and S10. (c) Comparison of all Fourier-transformed EXAFS data of the Pd_1_@C_3_N_4_ catalyst under different conditions described in Table S10 with a reference PdBr_2_ sample. The gray area and gray dashed line indicate the relevant scattering paths. (d) Simulated structures illustrating the coordination of palladium centers in Pd_1_@C_3_N_4_ upon adsorption of NaO^*t*^Bu, RuPhos, phenyl bromide substrate, or the effect of the ligand on the aryl halide adsorption. Color code: carbon, gray; nitrogen, blue; oxygen, red; palladium, green; phosphorus, yellow; bromide, brown; sodium, purple.

A broad range of reagent combinations was tested via in situ Pd *K*-edge XAS, including the isolated reaction components (ligands, bases, solvents), to assess the state of the catalyst upon interaction with them as well as under full reaction conditions (Figure 4a). The selected components were stirred at the reaction temperature (115 °C) for 2 h to let the catalytic system reach a steady state. For the experiment under full reaction conditions, 2 h is an appropriate reaction time to remain in the linear conversion range (Figure 2c) and prevent potential product inhibition or decomposition effects. Interestingly, only slight changes were observed in the XANES spectra over the different cases, indicating that the various reaction components cannot strongly change the electronic properties of the catalyst and that a state closely resembling a nominal Pd(II) oxidation state is adopted. Differentiation is likely also hampered to a certain extent by inherent XAS limitations such as the limit of detection, polydispersity and disorder effect, and averaging of all occurring Pd states.^64,65^ Nevertheless, a stepwise approach was used first to probe the capability of the various components to coordinate. With LiHMDS (SB1) or NaO^*t*^Bu (SB2) the whiteline intensity decreases or slightly increases, respectively, indicating coordination of the two components as well as partial reduction or oxidation as a result. Further evidence might be found in the derivative spectra (Figure S8) or corresponding descriptors (Table S1), where some changes may more easily be observed. In the derivative spectra, a new feature at 24,360 eV appears with a simultaneous reduction of the main feature at 24,350 eV. In the presence of RuPhos and either base (SB1L or SB2L), the competitive adsorption between the two on the palladium sites can be seen as an intermediate spectrum between ligand-only (SL) and base-only cases (SB1 or SB2). In fact, the coordination of LiHMDS with the palladium sites can be readily observed via ex situ Pd *L*_3_-edge (Figure S9), as an additional feature at 3178 eV in the XANES region. This feature disappears following the application of full reaction conditions, suggesting a limited importance of base coordination as excess base should otherwise have remained coordinated. The limited importance of base coordination with RuPhos matches previous observations of the SM coupling over the same catalyst, where RuPhos was shown to suppress negative side effects (e.g., selectivity loss) of competitive ligand-base coordination.^33^ Importantly, under all tested conditions EXAFS analysis shows that the first-shell coordination number (CN) does not exceed 4 (Table S10). Furthermore, as the first peak corresponding to Pd–C/N/O bonds shows no major deformation or evolution of shoulders, the formation of a predominant Pd–P environment seems unlikely, which would be expected if the metal center was displaced by the surrounding ligands. Accordingly, as the Pd atoms remain anchored to the carrier support throughout, the number of available coordination sites for the reaction remains limited and the catalytic cycle must include more ligand exchange steps. Additionally, many conditions lead to lower first-shell CN, likely caused by the movement of some species to the second shell which could not be analyzed due to insufficient spectral quality.

Before entering the catalytic cycle (cf. Section 3.5), commonly employed homogeneous Pd precatalysts need to undergo an activation step, wherein the Pd(II) atoms undergo a reduction to Pd(0).^48,50,66^ In some cases, the free ligand can act as a reducing agent.^67^ Performing a similar experiment over Pd_1_@C_3_N_4_ in the presence of RuPhos, no signs of reduction to Pd(0) are apparent from either XANES or EXAFS analyses. Instead, Pd(II) is prevalent under these conditions despite the phosphine excess (experiments 2, 8, 10, and 11 in Figures 4b,c and S7, see Table S10 for details of the conditions and data analysis). When strong bases are used that can potentially also coordinate too strongly with palladium, such as LiHMDS, features of Pd(0) can be seen (Figures 4b, S10 and S11, especially in combination with certain ligands e.g., PPh_3_ and P^*t*^Bu_3_·HBF_4_). This is attributed to either the coordination of palladium atoms with the ligands to form Pd(PPh_3_)_4_ and/or to the formation of nanoparticles, as evidenced by HAADF-STEM in some cases (Figure S5). It should be noted that even in this case, Pd(II) remains the most abundant species (Figure 3b).

This in-depth analysis of the different reaction compound combinations and their respective ratios (Figure S10), and their electronic and geometric effect on the palladium sites provides valuable insights concerning the optimal environment for obtaining practically relevant yields and stable operation. Based on this, it is apparent that for Pd_1_@C_3_N_4_, RuPhos is the ligand of choice since, unlike PPh_3_, it does not oxidize under reaction conditions (^31^P NMR, Figure S4)^51^ and maintains the atomic dispersion of palladium centers. These results highlight the importance of selecting ligands with tailored steric and oxidation-resistant properties. Similarly, NaO^*t*^Bu, a base of sufficient strength that is not strongly coordinated to the palladium site is preferred. Such bases can aid the selective transformation of the reactants without promoting side reactions, while maintaining the initial active site architecture. On the contrary, strongly coordinating bases, e.g., LiHMDS, should be avoided or the possibility for background reactivity and modification of the active site structure must be considered.

### 3.5 Mechanistic Implications

The widely accepted catalytic cycle of the BH reaction includes several key steps: oxidative addition, amine coordination and deprotonation, and reductive elimination.^5,50^ During oxidative addition, the aryl halide adds to the metal center reducing its oxidation state and forming a Pd-halide and Pd–C bond. This step is generally considered rate-limiting, resulting in the well-known halide reactivity trend (R–I > R–Br > R–Cl). Following this, the amine reagent coordinates with the Pd complex. Deprotonation leads to the formation of a halide salt, and the resulting complex undergoes reductive elimination of the amine and aryl ligand. The cycle often involves a Pd activation step, wherein Pd(II) precursors are reduced to Pd(0), and may include ligand exchange steps, especially when bidentate ligands are employed.^5^ As discussed in Section 3.4, we expect that additional ligand exchange steps would be required in the case of Pd_1_@C_3_N_4_ because the carbon nitride carrier occupies a significant fraction of the coordination sphere, in analogy to bidentate ligands.

The inability to identify nominal Pd(0), except in the case where Pd nanoparticle formation was observed, and the prevalence of Pd(II) in the case of Pd_1_@C_3_N_4_ is particularly intriguing (Figure 4b) considering the established mechanistic understanding for coupling reactions, including BH using homogeneous catalysts, where Pd(0) is typically viewed to be the active catalytic state and even the resting state of palladium depending on the conditions.^68^ Our analysis indicates an error of less than 2% for the cases where Pd(0) is absent (Table S11), although the formation of Pd(0) intermediates cannot be completely excluded given the limitations of XAS.^64,65^ The hypothesis that such a small fraction of the palladium sites could be solely responsible for the reactivity cannot be supported, especially when comparing yields for reaction conditions where nanoparticle formation and leaching were observed. One possible explanation is that Pd(0) centers are transient, short-lived species and that the XAS signal observed under full reaction conditions reflects the Pd atom in a different stage of the cycle. This would imply that Pd activation is driven by the subsequent catalytic cycle and ultimately the product formation, rather than an independent redox process. Alternatively, the semi-infinite pool of electrons and holes existing on the highly aromatic carbon nitride semiconductor host may hinder the adoption of distinct nominal oxidation states. Both scenarios imply that the working mechanisms differ compared to homogeneous catalytic systems.

Density functional theory (DFT) simulations corroborate changes in the electronic state of palladium sites as their coordination to the support adapts upon the adsorption of a ligand (Figure 4d, Table S12). Given the strong binding energy of the ligands to the palladium atom and the higher coordination of the latter with the support, we can deduce that the ligand plays a role in stabilizing the palladium centers on the surface of the carbon nitride heptazine ring. This is also demonstrated by the slight shift in the absorption edge position to higher energies as the amount of ligand is increased (Figure S11), indicating the protecting, stabilizing character of the ligand. The coordination of the ligand on the palladium sites was further corroborated via ex situ P *K*-edge XAS (Figure S12).^69,70^

Interestingly, DFT simulations (Table S12) show that the adsorption of the bromobenzene is slightly more favorable in the presence of adsorbed ligands on Pd_1_@C_3_N_4_ (−0.67 eV with RuPhos, −0.56 eV with PPh_3_, and −0.57 eV in the absence of ligand). Considering the experimental results, which highlight the need for a ligand in the reaction to achieve high product yields (Figure S3, Table S2), the values obtained from the simulations suggest that the ligand likely plays an activating role for palladium sites in the transition state or at later stages of the reaction. Indeed, both PPh_3_ and RuPhos stabilize palladium centers from the subsurface to surface positions within C_3_N_4_ (shift of 1.65 and 2.31 Å, respectively),^71^ rendering them accessible to participate in the reaction. Overall, the choice of the ligand defines the dynamic active site architecture, which comprises the interaction of palladium centers with two nitrogen atoms in the C_3_N_4_ host and one P atom from the ligand (Figure 4d). Both the XAS analysis and DFT simulations indicate that the latter might partially decoordinate during certain steps to make space for the reaction to occur, still retaining however its heterogeneous character. The requirement for multiple ligand-substitution steps agrees with mechanisms proposed for Pd complexes containing bidentate ligands.^5^

Mechanistically, it is evident that the conversion of the aryl halides is facile in the presence of electron-withdrawing groups. Combined with the easier activation of chlorides rather than iodides in these cases, the oxidative addition of the aryl halides should not be the rate-limiting step in the reaction. This differs from the behavior observed for organometallic complexes under comparable conditions.^45,72^ Furthermore, the EXAFS analysis does not show any sign of Pd–Br bond formation under any tested condition (Figure 4c), even in the presence of excess (10 equiv) bromobenzene. The DFT results indicate slight preferential adsorption of the C atom (−0.67 eV) over the Br atom (−0.61 eV) of the bromobenzene molecule to the palladium center (Table S12). While no quantitative analysis can be done for such small differences from DFT, qualitatively this result is consistent in all cases (i.e., ligand-free or with PPh_3_ or RuPhos). Furthermore, the coordination of the carbon atom in aryl benzenes has been previously reported as an intermediate step for homogeneous [(Ph_3_P)_2_Pd(dba)] catalysts based on XAS analysis,^73^ where a growing contribution of the Pd-halide bond was simultaneously observed. However, in the case of Pd_1_@C_3_N_4_, no sign of Pd-halide coordination was evident even after 2 h (Table S10, Note S1). This could be attributed either to a reaction mechanism in which the Pd–Br bond dissociation is so facile that it is not captured as an intermediate, or that another functionality of the catalyst (e.g., related to the host) is involved in the halogen removal in tandem with the base. Elemental maps of the used catalysts obtained via EDX evidence a uniform deposition of Br across the catalyst surface, suggesting a potential affinity of the halide for the C_3_N_4_ host. Precipitation of Na–X (X = Cl, Br, I) from the nonpolar toluene solvent is expected, followed by its deposition on the catalyst surface (Figure S13).^62^ Importantly, these deposits do not detriment the function and they can be readily removed by washing in different solvents (water, methanol, dioxane), pointing to the poisoning resistance of the catalyst.

We note that, in contrast to our investigations, theoretical analysis on the potential of Pd/TiO_2_ SACs to activate C–X (X = Cl, Br, I) bonds predicted a clear interaction between Pd and Br and attributed this to the unique electronic properties of palladium sites that permitted facile C–X bond activation.^74^ This deviation from experimental results, also observed in our DFT simulations, could arise from the simplifications used in modeling SACs in liquid-phase environments, which currently may still not account for the solvent.^75^ Our results further underline the importance of confirming theoretical simulations with experimental results. Taken together, our experimental and theoretical investigations of the Pd SAC point to a heterogeneous reaction mechanism that involves a palladium site stabilized on the carbon nitride host and coordinating with the RuPhos ligand during the catalytic cycle. The heterogeneous catalyst demonstrates promising activity, selectivity, and stability.

Practically, considering both the potential for reducing the metal carbon-footprint and the substantial contribution (ca. 30%)^32^ that aryl halides can have, SACs could offer a complementary tool to established homogeneously catalyzed processes, improving the sustainability of C–N coupling applications.^11^ This could be especially the case for the use of chlorides when their coupling is preferred over bromides or iodides from an environmental and financial point of view (Table S13). Furthermore, the availability of robust solid catalysts enables the use of fixed-bed reactors to facilitate the transition to continuous production and process intensification where suitable. Investigating the solvent effects and compatibility with biomass-derived solvents will be just as important for these goals for economic and environmental sustainability.

## 4 Conclusions

Exploring the potential of palladium SACs, our study demonstrated Pd_1_@C_3_N_4_ as an active, selective, and stable catalyst in various Buchwald–Hartwig aminations, particularly those involving primary and secondary amines, with the efficacy depending on the specific substrate combination. Notably, our results revealed complementary synthetic capabilities to commonly applied homogeneous catalytic systems, including a preference toward coupling distinct aryl chlorides over iodides in some reactions and a generally high chemoselectivity to amines over amides. In situ XAS investigations provided unprecedented insights into the structure and coordination of palladium atoms within the heptazinc cavities of the carbon nitride host during the reaction. The results highlighted distinct electronic and steric properties compared to traditional homogeneous systems, identifying a Pd(II) oxidation state across all steps of the reaction and highlighting the pivotal role of the ligand in activating and stabilizing the active metal centers. Furthermore, the lack of formation of a Pd–Br bond also points to mechanistic distinctions from soluble metal complex catalysts. While our study serves as a proof of concept in developing Pd SACs for Buchwald–Hartwig couplings, further exploration dedicated to optimizing the three-dimensional structures of SACs for efficiently synthesizing more complex substrates is required to elucidate the scope fully. Nonetheless, our results underscore significant potential for advancing the sustainable production of pharmaceuticals, agro, and fine chemicals through heterogeneous catalysis with SACs.

## Data Availability

All data obtained in this study is openly accessible through Zenodo: 10.5281/zenodo.10927466.

## Supporting Information Available

Characterization, catalytic, and analytical data (PDF)
