# Supplementary material for: Reactivity and Mechanism of Recoverable Pd1@C3N4 Single-Atom Catalyst in Buchwald–Hartwig Aminations
Source: ACS Catal. 2024 Dec 17;15(1):284–95. doi: 10.1021/acscatal.4c05134 (PMC11705219; doi:10.1021/acscatal.4c05134)
Supplement: Supplementary file 1 — cs4c05134_si_001.pdf [file cs4c05134_si_001.pdf]

Supporting Information

# Reactivity and Mechanism of Recoverable $Pd_1@C_3N_4$ Single-Atom Catalyst in Buchwald-Hartwig Aminations

*Georgios Giannakakis,[a] Marc Eduard Usteri,[a] Aram Bugaev,[b] Andrea Ruiz-Ferrando,[c,d] Dario Faust Akl,[a] Núria López,[c] Serena Fantasia,[e] Kurt Püntener,[e] Javier Pérez-Ramírez,*[a] Sharon Mitchell*[a]*

[a] Institute of Chemical and Bioengineering, Department of Chemistry and Applied Biosciences, ETH Zurich, Vladimir-Prelog-Weg, 1, 8093 Zurich, Switzerland

Sharon Mitchell. E-mail: sharon.mitchell@chem.ethz.ch

Javier Pérez-Ramírez. E-mail: jpr@chem.ethz.ch

[b] Paul Scherrer Institute, Forschungsstrasse 111, 5232 Villigen, Switzerland

[c] Institute of Chemical Research of Catalonia (ICIQ), The Barcelona Institute of Science and Technology, Av. Països Catalans 16, 43007 Tarragona, Spain

[d] University of Rovira i Virgili, Av. Catalunya 35, 43002 Tarragona, Spain

[e] Pharmaceutical Division, Synthetic Molecules Technical Development, Process Chemistry & Catalysis, F. Hoffmann-La Roche Ltd, 4070 Basel, Switzerland

**Contents**

Supplementary Tables 1
Supplementary Note S1 15
Supplementary Figures 16
Supplementary Data 1 25
Supplementary Note S2 25
Supplementary References 30

**Table S1.** Descriptors of the in-situ XANES and corresponding derivative spectra. The graphical interpretation of the descriptors is shown in **Figure S1**.

| Entry | System[a] | XANES[b] | | | Derivative spectra[b] | | |
|---|---|---|---|---|---|---|---|
| | | $E_{max}$ / eV | $H_{max}$ / a.u. | $D^2_{max}$ / $10^{-3}$ | $E_d$ / eV | $W_d$ / eV | $H_d$ / $10^{-3}$ a.u. |
| 1 | S | 24376.43 | 1.092 | 2.61 | 24355.94 | 17.98 | 55.19 |
| 2 | SL(1) | 24376.53 | 1.092 | 2.93 | 24356.05 | 19.01 | 54.11 |
| 3 | SB(1)L(1) | 24376.79 | 1.068 | 1.60 | 24356.35 | 28.08 | 50.31 |
| 4 | SB(1)L(1)R(1) | 24376.12 | 1.092 | 2.60 | 24356.78 | 24.75 | 51.66 |
| 5 | SB(1) | 24376.49 | 1.053 | 0.97 | 24355.88 | 45.51 | 48.54 |
| 6 | SL(2) | 24377.18 | 1.073 | 1.15 | 24355.35 | 18.23 | 53.23 |
| 7 | SB(2) | 24375.26 | 1.096 | 2.25 | 24356.77 | 28.60 | 50.03 |
| 8 | SB(2)L(1) | 24376.01 | 1.098 | 2.16 | 24356.95 | 25.50 | 50.80 |
| 9 | SB(1)L(1)R(1)R(2) | 24378.25 | 1.061 | 1.29 | 24356.46 | 29.55 | 50.42 |
| 10 | SL(1)[c] | 24375.14 | 1.086 | 1.28 | 24356.43 | 24.35 | 50.96 |
| 11 | SL(1)[d] | 24375.40 | 1.088 | 2.34 | 24356.63 | 25.00 | 51.02 |
| 12 | SB(1)[e] | 24375.17 | 1.096 | 1.89 | 24356.82 | 23.59 | 51.79 |
| 13 | SB(1)[f] | 24374.60 | 1.067 | 1.95 | 24355.51 | 38.50 | 47.94 |
| 14 | SB(1)L(2) | 24375.11 | 1.085 | 1.89 | 24356.04 | 26.90 | 50.53 |
| 15 | SB(1)L(3) | 24375.15 | 1.085 | 1.10 | 24356.69 | 29.72 | 48.85 |
| 16 | SB(2)L(2) | 24374.65 | 1.073 | 1.15 | 24355.56 | 56.52 | 47.15 |
| 17 | SB(2)L(3) | 24374.98 | 1.090 | 2.12 | 24356.07 | 25.02 | 51.11 |
| 18 | SR(2) | 24375.44 | 1.082 | 0.88 | 24355.91 | 21.33 | 52.31 |
| 19 | SL(1)R(2) | 24375.78 | 1.086 | 2.65 | 24356.09 | 23.72 | 51.05 |
| 20 | SB(1)R(2) | 24374.84 | 1.049 | 1.69 | 24355.04 | 60.33 | 46.38 |

[a] In-situ measurements studied the catalysts in mixtures containing the solvent (S, toluene) on its own or with other reaction components including the base after 2 h of heating at 115°C: B(1) - LiHMDS or B(2) - NaO$^t$Bu, ligand: L(1) - RuPhos, L(2) - $PPh_3$, or L(3) - $P^tBu_3 \cdot HBF_4$, and/or reactants: R(1) - bromobenzene or R(2) - morpholine. The codes indicate the reagents present in each measurement. Entry 9 corresponds to the full BH reaction conditions with L(1). Reaction conditions as described in **Table S2**. [b] Descriptors of the spectra as defined in **Figure S1**. [c] L:Pd = 1:1. [d] L:Pd = 1:10. [e] B:Pd = 1:1. [f] B:Pd = 1:3.

**Table S2.** Initial investigation of the $Pd_1@C_3N_4$ catalyst in the BH amination of bromobenzene and morpholine to phenylmoropholine.[a]

| Ligand | Ligand / mol% | Toluene | | Anisole | | DME:$H_2O$[b] | |
|---|---|---|---|---|---|---|---|
| | | NaO$^t$Bu | LiHMDS | NaO$^t$Bu | LiHMDS | NaO$^t$Bu | $K_2CO_3$ |
| $PPh_3$ | 5 | 41 | 52 | 0 | 57 | 0 | 0 |
| | 10 | 23 | 38 | 21 | 28 | 0 | 0 |
| $PCy_3$ | 5 | 11 | 37 | 9 | 48 | 0 | 0 |
| | 10 | 6 | 37 | 3 | 31 | 0 | 0 |
| $P^tBu_3 \cdot HBF_4$ | 5 | 24 | 43 | 3 | 52 | 0 | 0 |
| | 10 | 73 | 27 | 48 | 43 | 0 | 1 |
| RuPhos | 5 | 3 | 46 | 56 | 67 | 0 | 0 |
| | 10 | 56 | 55 | 55 | 55 | 0 | 0 |

[a] General reaction conditions: Pd (0.01 equiv), solvent (toluene, anisole, or DME: 1,2-dimethoxyethane and $H_2O$), base (NaO$^t$Bu, LiHMDS, or $K_2CO_3$, 3 equiv), ligand ($PPh_3$, $PCy_3$, $P^tBu_3 \cdot HBF_4$, or RuPhos, 5 or 10 mol%), bromobenzene (1 equiv), morpholine (1.5 equiv), $T$ = 115°C, $t$ = 2 h. Reported values correspond to area % product formation determined by LC-MS. [b] DME and water were mixed in a volume-based 3:1 ratio.

**Table S3.** Quantification of Pd content in solutions obtained after treatment of $Pd_1@C_3N_4$ with different solvent, base, and ligand combinations.

| Reaction[a] | Ligand | Base | Solvent | $Pd_{sol}$ / ppm[b] |
|---|---|---|---|---|
| 1 | RuPhos | NaO$^t$Bu | Toluene | 0.09 |
| 2 | $PPh_3$ | NaO$^t$Bu | Toluene | 1.09 |
| 3 | $PPh_3$ | NaO$^t$Bu | Anisole | 1.14 |
| 4 | $PPh_3$ | LiHMDS | Anisole | 1.45 |
| 5 | $PCy_3$ | NaO$^t$Bu | Anisole | 1.86 |
| 6 | $PCy_3$ | LiHMDS | Anisole | 0.55 |
| 7 | $P^tBu_3 \cdot HBF_4$ | NaO$^t$Bu | Anisole | 4.11 |
| 8 | $P^tBu_3 \cdot HBF_4$ | LiHMDS | Anisole | 1.41 |

[a] Following the general reaction conditions described in **Table S2**. [b] Palladium content in solution, determined by ICP-OES.

**Table S4.** Extended scope of $Pd_1@C_3N_4$-catalyzed BH amination over 4 h using LiHMDS as the base.[a]

| | 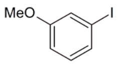 | | 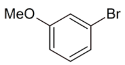 | | 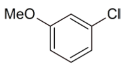 | | 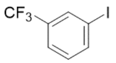 | | 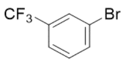 | | 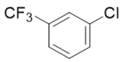 | | 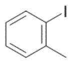 | | 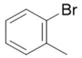 | | 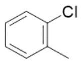 | |
|---|---|---|---|---|---|---|---|---|---|---|---|---|---|---|---|---|---|---|
| | +c | -c | +c | -c | +c | -c | +c | -c | +c | -c | +c | -c | +c | -c | +c | -c | +c | -c[b] |
| 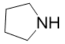 | 35 | 0 | 61 | 0 | 85 | 0 | 79 | 0 | 92 | 49 | 84 | 0 | 38 | 0 | 50 | 0 | 44 | 9 |
| 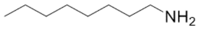 | 40 | 0 | 46 | 11 | 43 | 0 | 47 | 7 | 57 | 18 | 60 | 13 | 83 | 0 | 91 | 0 | 95 | 0 |
| 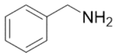 | 57 | 0 | 44 | 17 | 40 | 0 | 56 | 0 | 40 | 31 | 67 | 8 | 76 | 0 | 77 | 83 | 80 | 0 |
| 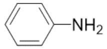 | 100 | 24 | 100 | 9 | 74 | 24 | 98 | 0 | 97 | 0 | 78 | 0 | 97 | 4 | 97 | 0 | 72 | 6 |
| 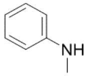 | 91 | 31 | 24 | 31 | 11 | 37 | 5 | 2 | 30 | 0 | 41 | 0 | 89 | 0 | 78 | 0 | 11 | 0 |
| 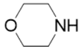 | 73 | 90 | 91 | 100 | 82 | 98 | 68 | 80 | 87 | 89 | 85 | 95 | 73 | 14 | 89 | 15 | 74 | 55 |
| 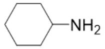 | 19 | 0 | 27 | 0 | 22 | 0 | 73 | 0 | 62 | 10 | 68 | 0 | 38 | 0 | 59 | 0 | 65 | 0 |
| 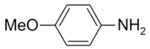 | 64 | 0 | 95 | 0 | 19 | 0 | 70 | 0 | 15 | 0 | 46 | 0 | 94 | 0 | 96 | 0 | 29 | 0 |

[a] Performed following the standardized high-throughput BH testing protocol with catalyst (+c) using RuPhos ligand and LiHMDS base, $t = 4$ h. Reported values correspond to area % product formation determined by LC-MS. The background activity was evaluated by testing in the absence (-c) of the catalyst for 4 or 20 h.

**Table S5.** Extended scope of $Pd_1@C_3N_4$-catalyzed BH amination over 20 h using LiHMDS as the base.[a]

| | 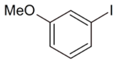 | | 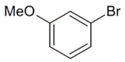 | | 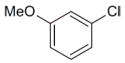 | | 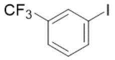 | | 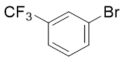 | | 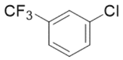 | | 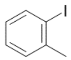 | | 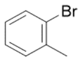 | | 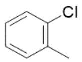 | |
|---|---|---|---|---|---|---|---|---|---|---|---|---|---|---|---|---|---|---|
| | +c | -c | +c | -c | +c | -c | +c | -c | +c | -c | +c | -c | +c | -c | +c | -c | +c | -c |
| 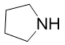 | 38 | 0 | 56 | 0 | 87 | 0 | 82 | 0 | 91 | 0 | 89 | 0 | | 0 | 52 | 6 | 26 | 9 |
| 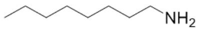 | 44 | 0 | 48 | 0 | 54 | 0 | 46 | 7 | 64 | 10 | 62 | 13 | 88 | 0 | 88 | 0 | 88 | 0 |
| 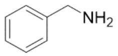 | 57 | 0 | 43 | 6 | 43 | 0 | 54 | 0 | 38 | 0 | 67 | 8 | 75 | 0 | 75 | 0 | 80 | 0 |
| 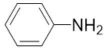 | 94 | 24 | 96 | 9 | 56 | 24 | 94 | 0 | 92 | 0 | 86 | **0** | **96** | **4** | **93** | **5** | **79** | **6** |
| 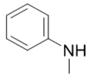 | 90 | 31 | 81 | 38 | 13 | 37 | **94** | 2 | **88** | 0 | **89** | 0 | 86 | 0 | 80 | 0 | 14 | 0 |
| 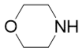 | 72 | 90 | 90 | 96 | 81 | 98 | 67 | 80 | 94 | 92 | 88 | **95** | **68** | 14 | 88 | 51 | 65 | 55 |
| 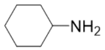 | 19 | 0 | 26 | 0 | 23 | 0 | 78 | 0 | 70 | 0 | 82 | 0 | 71 | 0 | **68** | **0** | **57** | 0 |
| 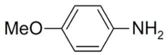 | 89 | 0 | 89 | 0 | 16 | 0 | 79 | 0 | 56 | 0 | 40 | 0 | 89 | 0 | 89 | 0 | 45 | 0 |
| 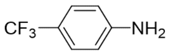 | 0 | 0 | 0 | 0 | 0 | 0 | 0 | 0 | 0 | 0 | 0 | 0 | 0 | 0 | 0 | 0 | 0 | 0 |

[a] Performed following the standardized high-throughput BH testing protocol with catalyst (+c) using RuPhos ligand and LiHMDS base, $t$ = 20 h. Reported values correspond to area % product formation determined by LC-MS. The background activity was evaluated by testing in the absence (-c) of the catalyst for 20 h. Yields in bold verified by $^1$H NMR with an internal standard (**Note S2**), selected spectra shown in **Figures S12-S19**.

**Table S6.** Scope of $Pd_1@C_3N_4$ catalyzed BH amination over 20 h using NaO$^t$Bu as the base.[a]

| | 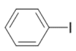 | 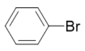 | 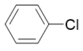 | 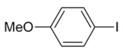 | 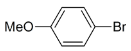 | 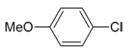 | 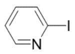 | 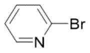 | 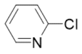 | 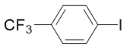 | 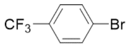 | 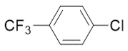 |
|---|---|---|---|---|---|---|---|---|---|---|---|---|
| 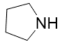 | 24 | 20 | 12 | 3 | 7 | 1 | 24 | 31 | 39 | 13 | 29 | 27 |
| 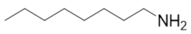 | 65 | 10 | 22 | 34 | 28 | 7 | 45 | 81 | 70 | 4 | 5 | 6 |
| 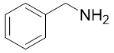 | 91 | -[b] | 28 | 51 | 21 | 3 | 35 | 31 | 64 | 0 | 0 | 0 |
| 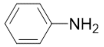 | -[b] | 95 | 9 | 94 | 80 | 17 | 84 | 90 | 85 | 4 | 0 | 0 |
| 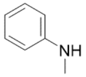 | 72 | 91 | 40 | 75 | 66 | 13 | 33 | 39 | 42 | 83 | 93 | 92 |
| 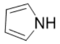 | 0 | 0 | 0 | 0 | 0 | 0 | 0 | 0 | 0 | 0 | 0 | 0 |
| 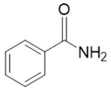 | 0 | 0 | 0 | 0 | 0 | 0 | 4 | 13 | 7 | 0 | 0 | 0 |
| 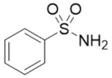 | 0 | 0 | 0 | 0 | 0 | 0 | 0 | 0 | 0 | 0 | 0 | 0 |

[a] Performed following the standardized high-throughput BH testing protocol using RuPhos ligand and NaO$^t$Bu base $t = 20$ h. Reported values correspond to area % product formation determined by LC-MS. [b] Not possible to quantify due to product/reactant overlap.

**Table S7.** Substrate conversion for scope experiments of $Pd_1@C_3N_4$ catalyzed BH amination over 20 h using NaO$^t$Bu as the base.[a]

| | 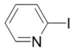 | 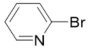 | 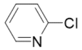 | 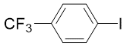 | 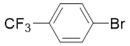 | 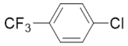 |
|---|---|---|---|---|---|---|
| 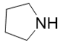 | 29 | 46 | 45 | 17 | 38 | 53 |
| 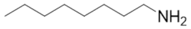 | 45 | 81 | 73 | 36 | 56 | 80 |
| 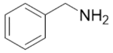 | 87 | 85 | 85 | 40 | 36 | 84 |
| 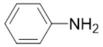 | 87 | 97 | 85 | 48 | 68 | 77 |
| 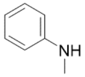 | 40 | 52 | 53 | 87 | 97 | 96 |
| 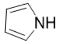 | 3 | 5 | 13 | 2 | 5 | 25 |
| 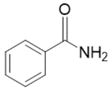 | 4 | 13 | 7 | 0 | 6 | 25 |
| 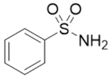 | 0 | 0 | 6 | 0 | 0 | 0 |

[a] Performed following the standardized high-throughput BH testing protocol using RuPhos ligand and NaO$^t$Bu base $t = 20$ h. Reported values correspond to area % substrate determined by LC-MS.

**Table S8.** Turnover frequency (*TOF*) reported for BH coupling over heterogeneous catalysts in literature and this work for comparable reactions.

| Entry[a] | Catalyst | Ligand | Base | Solvent | Aryl halide | Amine | $T$ / °C | $t$ / h | Yield / % | $TOF$ / $h^{-1}$ | Ref |
|---|---|---|---|---|---|---|---|---|---|---|---|
| 1 | $SiO_2$@green tea/Pd | none | $Cs_2CO_3$ | DMF | 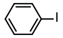 | 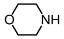 | 100 | 12 | 96 | 16.0 | [1] |
| 2 | GO-Chit-Pd | none | *t*-BuOK | DMF | 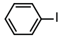 | 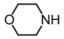 | 100 | 12 | 96 | 16.0 | [2] |
| 3 | PNP-SSS | none | $K_2CO_3$ | DMF | 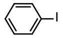 | 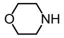 | 120 | 4 | 99 | 41.3 | [3] |
| 4 | nano PdAu | none | KO$^t$Bu | DMSO | 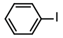 | 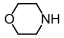 | 100 | 12 | 91 | 2.5 | [4] |
| 5 | $SiO_2$@green tea/Pd | none | $Cs_2CO_3$ | DMF | 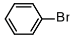 | 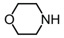 | 100 | 12 | 95 | 15.8 | [1] |
| 6 | GO-Chit-Pd | none | *t*-BuOK | DMF | 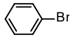 | 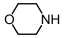 | 100 | 12 | 90 | 15.0 | [2] |
| 7 | PNP-SSS | none | $K_2CO_3$ | DMF | 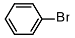 | 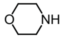 | 120 | 6 | 97 | 26.9 | [3] |
| 8 | nano PdAu | none | KO$^t$Bu | DMSO | 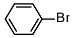 | 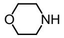 | 100 | 12 | 93 | 2.6 | [4] |
| 9 | PFG-Pd | none | NaO$^t$Bu | toluene | 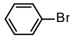 | 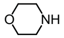 | 110 | 12 | 90 | 4.4 | [5] |
| 10 | PFG-Pd | none | NaO$^t$Bu | toluene | 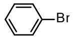 | 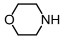 | 110 | 12 | 88 | 4.3 | [5] |
| 11 | Pd black | none | KO$^t$Bu | 1,4-dioxane | 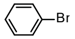 | 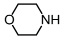 | 100 | 24 | 15 | 4.6 | [6] |
| 12 | Pd/C | none | KO$^t$Bu | 1,4-dioxane | 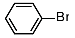 | 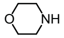 | 100 | 24 | 21 | 5.7 | [6] |
| 13 | $SiO_2$@green tea/Pd | none | $Cs_2CO_3$ | DMF | 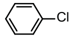 | 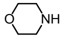 | 100 | 12 | 60 | 10.0 | [1] |
| 14 | GO-Chit-Pd | none | *t*-BuOK | DMF | 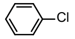 | 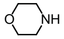 | 100 | 12 | 58 | 9.7 | [2] |
| 15 | PNP-SSS | none | $K_2CO_3$ | DMF | 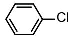 | 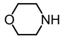 | 120 | 24 | 76 | 5.3 | [3] |
| 16 | nano PdAu | none | KO$^t$Bu | DMSO | 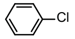 | 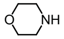 | 100 | 12 | 95 | 2.6 | [4] |
| 17 | nano PdAu | none | KO$^t$Bu | DMSO | 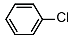 | 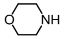 | 100 | 12 | 96 | 2.7 | [4] |

| | | | | | | | | | | | |
|---|---|---|---|---|---|---|---|---|---|---|---|
| 18 | $SiO_2$@green tea/Pd | none | $Cs_2CO_3$ | DMF | 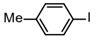 | 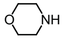 | 100 | 12 | 96 | 16.0 | [1] |
| 19 | PNP-SSS | none | $K_2CO_3$ | DMF | 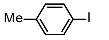 | 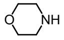 | 120 | 6 | 91 | 25.3 | [3] |
| **20** | **$Pd_1$@$C_3N_4$** | **RuPhos** | **LiHMDS** | **toluene** | 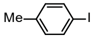 | 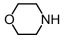 | **115** | **4** | **73** | **18.3** | **-** |
| 21 | $SiO_2$@green tea/Pd | none | $Cs_2CO_3$ | DMF | 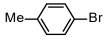 | 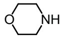 | 100 | 12 | 90 | 15.0 | [1] |
| 22 | GO-Chit-Pd | none | *t*-BuOK | DMF | 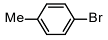 | 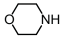 | 100 | 12 | 98 | 16.3 | [2] |
| 23 | PNP-SSS | none | $K_2CO_3$ | DMF | 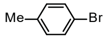 | 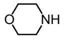 | 120 | 12 | 89 | 12.4 | [3] |
| **24** | **$Pd_1$@$C_3N_4$** | **RuPhos** | **LiHMDS** | **toluene** | 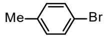 | 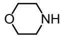 | **115** | **4** | **89** | **22.3** | **-** |
| 25 | PNP-SSS | none | $K_2CO_3$ | DMF | 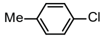 | 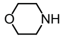 | 120 | 24 | 55 | 3.8 | [3] |
| 26 | nano PdAu | none | $KO^tBu$ | DMSO | 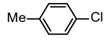 | 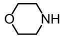 | 100 | 12 | 89 | 2.5 | [4] |
| 27 | $SiO_2$@green tea/Pd | none | $Cs_2CO_3$ | DMF | 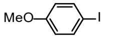 | 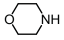 | 100 | 12 | 90 | 15.0 | [1] |
| 28 | GO-Chit-Pd | none | *t*-BuOK | DMF | 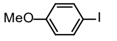 | 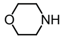 | 100 | 12 | 98 | 16.3 | [2] |
| 29 | $SiO_2$@green tea/Pd | none | $Cs_2CO_3$ | DMF | 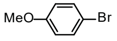 | 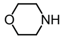 | 100 | 12 | 85 | 14.2 | [1] |
| 30 | GO-Chit-Pd | none | *t*-BuOK | DMF | 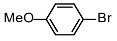 | 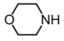 | 100 | 12 | 90 | 15.0 | [2] |
| 31 | nano PdAu | none | $KO^tBu$ | DMSO | 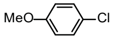 | 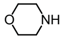 | 100 | 12 | 91 | 2.5 | [4] |
| 33 | Au-Pd | none | $KO^tBu$ | DMF | 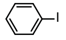 | 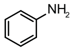 | 45 | 24 | 30 | 1.8 | [7] |
| 35 | Pd | none | $KO^tBu$ | DMF | 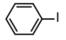 | 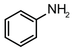 | 45 | 24 | 0 | 0 | [7] |
| 36 | nano PdAu | none | $KO^tBu$ | DMSO | 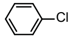 | 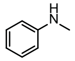 | 100 | 12 | 87 | 2.4 | [4] |
| 37 | PFG-Pd | none | $NaO^tBu$ | toluene | 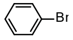 | 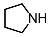 | 110 | 15 | 88 | 3.5 | [5] |

[a] Entries in bold indicate the results of the current study.

**Table S9.** Turnover frequency (*TOF*) reported for BH coupling over homogeneous catalysts in literature and this work for comparable reactions.

| Entry[a] | Catalyst | Ligand | Base | Solvent | Aryl halide | Amine | *T* / ºC | *t* / h | Yield / % | *TOF* / $h^{-1}$ | Ref |
|---|---|---|---|---|---|---|---|---|---|---|---|
| 1 | [Pd(L)Br($PPh_3$)] | 1-naphthaldehyde thiosemicarbazone | $K_2CO_3$ | 2-BuOH | 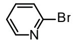 | 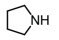 | 100 | 24 | 87 | 18.1 | [8] |
| 2 | [Pd(L)Br($PPh_3$)] | 1-naphthaldehyde thiosemicarbazone | $K_2CO_3$ | 2-BuOH | 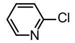 | 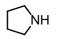 | 100 | 24 | 71 | 11.8 | [8] |
| 3 | Pd(Ar)Cl | RuPhos precatalyst | NaO*t*Bu | THF | 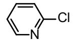 | 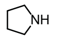 | 85 | 12 | 87 | 8.3 | [9] |
| 5 | [Pd(L)Br($PPh_3$)] | 1-naphthaldehyde thiosemicarbazone | $K_2CO_3$ | 2-BuOH | 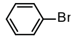 | 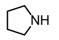 | 100 | 24 | 91 | 18.9 | [8] |
| 6 | [Pd(L)Br($PPh_3$)] | 1-naphthaldehyde thiosemicarbazone | $K_2CO_3$ | 2-BuOH | 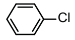 | 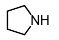 | 100 | 24 | 75 | 11.8 | [8] |
| 7 | [Pd(L)Br($PPh_3$)] | 1-naphthaldehyde thiosemicarbazone | $K_2CO_3$ | 2-BuOH | 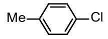 | 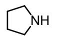 | 100 | 24 | 86 | 17.8 | [8] |
| **8** | **$Pd_1$@$C_3N_4$** | **RuPhos** | **LiHMDS** | **toluene** | 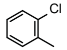 | 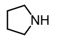 | **115** | **4** | **44** | **11.0** | **-** |
| 9 | [Pd(L)Br($PPh_3$)] | 1-naphthaldehyde thiosemicarbazone | $K_2CO_3$ | 2-BuOH | 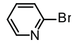 | 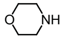 | 100 | 24 | 84 | 17.6 | [8] |
| 10 | [Pd(L)Br($PPh_3$)] | 1-naphthaldehyde thiosemicarbazone | $K_2CO_3$ | 2-BuOH | 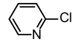 | 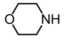 | 100 | 24 | 68 | 11.4 | [8] |
| 11 | [Pd(L)Br($PPh_3$)] | 1-naphthaldehyde thiosemicarbazone | $K_2CO_3$ | 2-BuOH | 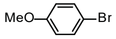 | 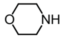 | 100 | 24 | 77 | 16.1 | [8] |
| 12 | Pd | RuPhos | NaO*t*Bu | THF | 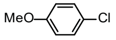 | 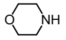 | 85 | 4 | 95 | 237.5 | [9] |
| 13 | [Pd(L)Br($PPh_3$)] | 1-naphthaldehyde thiosemicarbazone | $K_2CO_3$ | 2-BuOH | 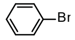 | 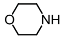 | 100 | 24 | 89 | 18.6 | [8] |
| 14 | Pd($PPh_3$)$_4$ | none | KO*t*Bu | 1,4-dioxane | 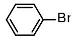 | 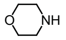 | 100 | 24 | 12 | 0.5 | [6] |
| 15 | $PdCl_2$ | none | KO*t*Bu | 1,4-dioxane | 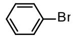 | 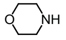 | 100 | 24 | 15 | 0.6 | [6] |
| 16 | $K_2PdCl_4$ | none | KO*t*Bu | 1,4-dioxane | 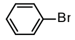 | 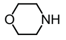 | 100 | 24 | 20 | 0.8 | [6] |
| 17 | Pd($pp_3S_4$)(dba) | none | $Cs_2CO_3$ | isopropanol | 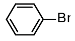 | 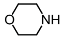 | 80 | 24 | 80 | 3.3 | [10] |
| 18 | [Pd(L)Br($PPh_3$)] | 1-naphthaldehyde thiosemicarbazone | $K_2CO_3$ | 2-BuOH | 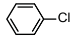 | 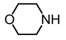 | 100 | 24 | 73 | 11.4 | [8] |

| | | | | | | | | | | | |
|---|---|---|---|---|---|---|---|---|---|---|---|
| 19 | Pd(pp$_3$S$_4$)(dba) | none | $Cs_2CO_3$ | isopropanol | 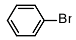 | 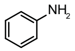 | 80 | 24 | 78 | 3.3 | [10] |
| 20 | 1% Pd(OAc)$_2$ | ClickPhos | KO$^t$Bu | toluene | 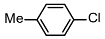 | 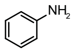 | 80 | 24 | 87 | 3.6 | [11] |
| 21 | 1% Pd(dba)$_2$ | ClickPhos | KO$^t$Bu | toluene | 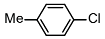 | 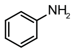 | 80 | 24 | 92 | 3.8 | [11] |
| 22 | 0.5% Pd(dba)$_2$ | ClickPhos | NaO$^t$Bu | toluene | 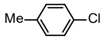 | 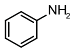 | 110 | 24 | 95 | 7.9 | [11] |
| 23 | 0.5% Pd(dba)$_2$ | ClickPhos | NaO$^t$Bu | toluene | 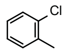 | 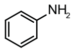 | 110 | 20 | 94 | 9.5 | [11] |
| **24** | **Pd$_1$@C$_3$N$_4$** | **RuPhos** | **LiHMDS** | **toluene** | 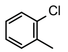 | 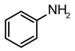 | **115** | **4** | **72** | **18.1** | **-** |
| 25 | Pd | RuPhos | NaO$^t$Bu | THF | 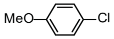 | 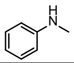 | 85 | 4 | 99 | 247.5 | [9] |

[a] Entries in bold correspond to results of the current study.

**Table S10.** EXAFS fitting parameters from ex-situ (fresh $Pd_1@C_3N_4$) and in-situ ($Pd_1@C_3N_4$ in solutions containing distinct solvent, base, ligand, and reactant combinations) measurements.

| Entry | System[a] | $R^b$ / Å | Coordination number / - | $\sigma^{2b}$ / $Å^2$ | $R$-factor / - |
|---|---|---|---|---|---|
| fresh | $Pd_1@C_3N_4$ | 2.09(3) | 4.4(1.4) | 0.004(4) | 0.08 |
| 1 | S | 2.03(2) | 4.1(9) | 0.006(4) | 0.04 |
| 2 | SL(1) | 2.03(2) | 4.1(9) | 0.006(4) | 0.04 |
| 3 | SB(1)L(1) | 2.00(1) | 2.7(3) | 0.002(1) | 0.01 |
| 4 | SB(1)L(1)R(1) | 1.99(2) | 3.2(5) | 0.002(2) | 0.02 |
| 5 | SB(1) | 1.99(1) | 2.5(3) | 0.003(2) | 0.02 |
| 6 | SL(2) | 2.04(3) | 3.9(1.0) | 0.007(5) | 0.07 |
| 7 | SB(2) | 2.00(2) | 3.2(5) | 0.002(2) | 0.02 |
| 8 | SB(2)L(1) | 1.99(2) | 3.2(6) | 0.001(2) | 0.03 |
| 9 | SB(1)L(1)R(1)R(2) | 1.99(1) | 2.7(3) | 0.002(1) | 0.02 |
| 10 | SL(1)[c] | 1.98(2) | 2.5(5) | 0.001(2) | 0.04 |
| 11 | SL(1)[d] | 2.00(2) | 2.9(6) | 0.001(2) | 0.04 |
| 12 | SB(1)[e] | 1.98(3) | 3.6(1.0) | 0.004(4) | 0.09 |
| 13 | SB(1)[f] | 2.00(1) | 2.5(4) | 0.003(2) | 0.02 |
| 14 | SB(1)L(2) | 1.97(2) | 2.7(5) | 0.001(2) | 0.03 |
| 15 | SB(1)L(3) | --[g] | | | |
| 16 | SB(2)L(2) | 1.99(1) | 2.3(3) | 0.001(1) | 0.02 |
| 17 | SB(2)L(3) | 2.00(1) | 2.3(4) | 0.001(2) | 0.04 |
| 18 | SR(2) | 2.00(1) | 2.6(3) | 0.001(1) | 0.01 |
| 19 | SL(1)R(2) | --[c] | | | |
| 20 | SB(1)R(2) | 1.99(2) | 1.8(3) | 0.001(2) | 0.04 |

[a] In-situ measurements studied the catalysts in mixtures containing the solvent (S, toluene) on its own or with other reaction components including the base after 2 h of heating at 115°C: B(1) - LiHMDS or B(2) - NaO$^t$Bu, ligand: L(1) - RuPhos, L(2) - $PPh_3$, or L(3) - $P^tBu_3 \cdot HBF_4$, and/or reactants: R(1) - bromobenzene or R(2) - morpholine. The codes indicate the reagents present in each measurement. Entry 9 corresponds to the full BH reaction conditions with L Reaction conditions as described in **Table S2**. [b] Fourier-transform parameters as outlined in **Note S1**. [c] L:Pd = 1:1. [d] L:Pd = 1:10. [e] B:Pd = 1:1. [f] B:Pd = 1:3. [g] Insufficient signal-to-noise ratio to fit spectra in EXAFS regime. This could result from a non-uniform catalyst distribution inside the vial or the impact of the stirring bar on the quality.

**Table S11.** Pd(0) content including the  as determined by linear fitting combination analysis of the in-situ XAS measurements

| Entry | System[a] | Pd(0) fraction / % |
|---|---|---|
| 1 | S | 0.0 ± 1.8 |
| 2 | SL(1) | 0.0 ± 1.8 |
| 3 | SB(1)L(1) | 11.1 ± 2.1 |
| 4 | SB(1)L(1)R(1) | 0.0 ± 2.2 |
| 5 | SB(1) | 22.2 ± 2.0 |
| 6 | SL(2) | 12.0 ± 1.8 |
| 7 | SB(2) | 0.0 ± 2.2 |
| 8 | SB(2)L(1) | 0.0 ± 2.4 |
| 9 | SB(1)L(1)R(1)R(2) | 15.6 ± 2.6 |
| 10 | SL(1)[c] | 0.0 ± 1.8 |
| 11 | SL(1)[d] | 0.0 ± 1.9 |
| 12 | SB(1)[e] | 0.0 ± 2.1 |
| 13 | SB(1)[f] | 19.1 ± 2.3 |
| 14 | SB(1)L(2) | 4.8 ± 1.8 |
| 15 | SB(1)L(3) | 2.4 ± 2.3 |
| 16 | SB(2)L(2) | 17.1 ± 2.1 |
| 17 | SB(2)L(3) | 4.0 ± 1.9 |
| 18 | SR(2) | 7.0 ± 1.7 |
| 19 | SL(1)R(2) | 5.0 ± 1.8 |
| 20 | SB(1)R(2) | 33.3 ± 2.4 |

[a] In-situ measurements studied the catalysts in mixtures containing the solvent (S, toluene) on its own or with other reaction components including the base after 2 h of heating at 115°C: B(1) - LiHMDS or B(2) - NaO$^t$Bu, ligand: L(1) - RuPhos, L(2) - $PPh_3$, or L(3) - $P^tBu_3 \cdot HBF_4$, and/or reactants: R(1) - bromobenzene or R(2) - morpholine. The codes indicate the reagents present in each measurement. Entry 9 corresponds to the full BH reaction conditions with L Reaction conditions as described in **Table S2**.

**Table S12.** DFT calculated binding energy values of solvents (THF, toluene), bases (NaO$^t$Bu, LiHMDS), ligands ($PPh_3$ or RuPhos), reactants (morpholine, bromobenzene - PhBr) and combinations with respect to the bare $Pd_1@C_3N_4$ structure.

| System | $E_{binding}$ / eV |
|---|---|
| $Pd_1@C_3N_4$-NaO$^t$Bu | −2.03 |
| $Pd_1@C_3N_4$-PhBr(Br)[a] | −0.50 |
| $Pd_1@C_3N_4$-PhBr(C)[b] | −0.57 |
| $Pd_1@C_3N_4$-LiHMDS | −2.76 |
| $Pd_1@C_3N_4$-LiHMDS-PhBr(Br)[a] | −0.04 |
| $Pd_1@C_3N_4$-LiHMDS-PhBr(C)[b] | −0.01 |
| $Pd_1@C_3N_4$-$PPh_3$ | −2.59 |
| $Pd_1@C_3N_4$-$PPh_3$-NaO$^t$Bu[c] | −1.23 |
| $Pd_1@C_3N_4$-$PPh_3$-PhBr(Br)[a] | −0.39 |
| $Pd_1@C_3N_4$-$PPh_3$-PhBr(C)[b] | −0.56 |
| $Pd_1@C_3N_4$-RuPhos[d] | −3.27 |
| $Pd_1@C_3N_4$-RuPhos-PhBr(Br)[a,d] | −0.61 |
| $Pd_1@C_3N_4$-RuPhos-PhBr(C)[b,d] | −0.67 |

[a] PhBr adsorbed on the $Pd_1@C_3N_4$(-L) structure via the Br.

[b] PhBr adsorbed on the $Pd_1@C_3N_4$(-L) structure via the ipso-C.

[c] NaO$^t$Bu coadsorbed on the $Pd_1@C_3N_4$-L structure.

[d] Simulations of RuPhos use a proxy replacing the isopropyl group by a hydrogen atom.

**Table S13.** Prices and global warming potentials (GWP) of selected aromatic halides.

| Halide | Price[a] / CHF $g^{-1}$ | GWP[b] / $kg_{CO2}$-eq |
|---|---|---|
| Chlorobenzene | 0.02-1.36 | 3.96 |
| Bromobenzene | 0.04-5.42 | 12.5 |
| Iodobenzene | 0.94-11.5 | 9.74 |
| 4-Chloroanisole | 2.35-62.8 | 96.3 |
| 4-Bromoanisole | 0.34-35.7 | 13.8 |
| 4-Iodoanisole | 1.32-52.7 | 33.5 |
| 4-Chlorotoluene | 0.72 | 66.5 |
| 4-Bromotoluene | 0.44-10.5 | 21.9 |
| 4-Iodotoluene | 2.12-2.54 | 18.2 |
| 4-Chloronitrobenzene | 0.30 | 23.3 |
| 4-Bromonitrobenzene | 4.64-5.16 | 43.2 |
| 4-Iodonitrobenzene | 2.80-3.57 | 37.7 |

[a] Prices from Sigma Aldrich website retrieved on 22/02/2024. Ranges reflect differences in purity and quantity. [b] GWP values given in [12].

**Note S1 | Comment on EXAFS data.** It was possible to measure $Pd_1@C_3N_4$ ex-situ in long *k*-range at high quality, which enabled observation of a double contribution directly in the R Space (inset **Figure 1c**). A possible reason for such a double contribution in $Pd_1@C_3N_4$ could be the adsorption of water because the samples are measured in air and there is no difference between dry and wet samples in the XANES region. Another reason could be the contribution from more distant N atoms in the $C_3N_4$ host. Analyzed in an identical way (in short 2.5 – 11.5 Å$^{-1}$ *k*-region), fit results are shown in **Table S10**. Higher Debye-Waller parameter and bad R-factor for all entries is the result of using a single shell, instead of the two. In addition, in $Pd_1@C_3N_4$ the presence of Cl, confirmed by the analysis of a longer EXAFS range, results in a significant increase of the first shell distance to ca. 2.1, compared to ca. 2.0 typical for Pd-C/N/O.

Based on such indirect indications, entries 1, 2, and 6 were spotted as potential samples with additional Pd-*X* contribution (*X* = Cl, P, or Br), due to increased first-shell distance, increased Debye-Waller, and relatively high R-factor. The addition of Pd-Br path leads to its negative amplitude in all three cases, so Pd-Br interaction can be excluded. The addition of Cl results in coordination numbers <0.4 in all cases and unphysically small or negative Debye-Waller factors. Obviously, the errors and correlations between parameters increase due to a higher number of variables. So, potentially these three samples may have some minor contribution of Cl. On the contrary, the lower first-shell distance, Debye-Waller, and R-factor values in all other cases (3, 4, 5, 7, 8, 9) point to the presence of a common component, which is the base. Therefore, it can be drawn that the strong Pd-Cl bonds can only be interrupted by the presence of the base. It should be noted that the rest of the tests listed in **Table S10** (10 through 20) are performed on a catalyst prepared by different palladium precursor (tetraamminepalladium(II) nitrate) to remove the effect of Cl for subsequent studies (ligand-base effects).

The almost negligible changes in first shell coordination distance might seem to give rise to a seeming contradiction with the DFT simulation, which predicts an extraction of the Pd atom out of its plane. If this movement were assumed to occur purely along the z-axis, prolongation of *R* would be expected. However, as the Pd atoms are not coordinated with all N atoms in a heptazine cavity and are thus not at its center, the extraction can follow a circular path with a constant radius. Accordingly, no changes in *R* values are needed, agreeing with the experimental results.

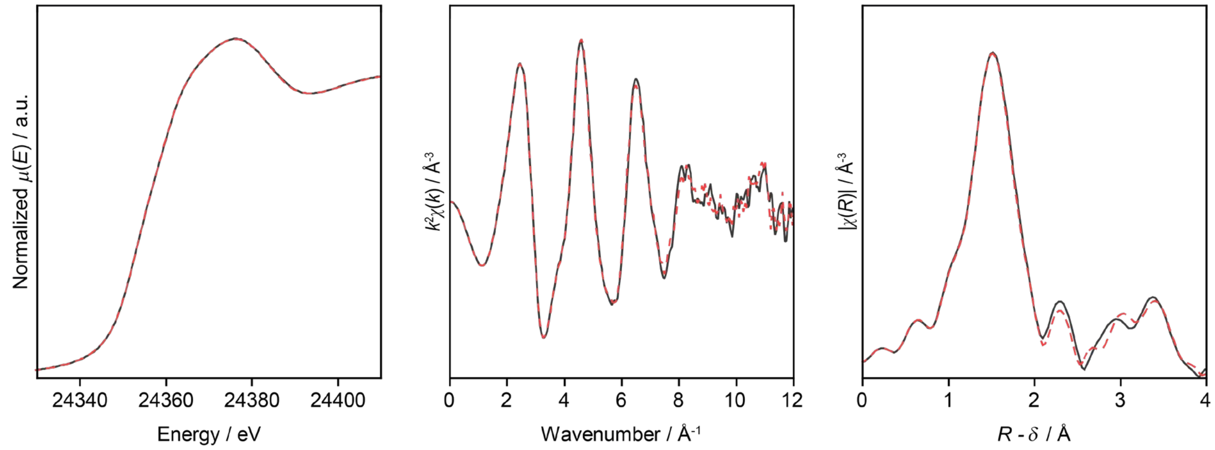


**Figure S1.** Averaged XANES and corresponding EXAFS spectra using the in-situ signal of $Pd_1@C_3N_4$ in pure solvent (BH-1). The black lines correspond to the average over the first 5 minutes and the red dashed lines to the average of the last 5 minutes of the measurement time.

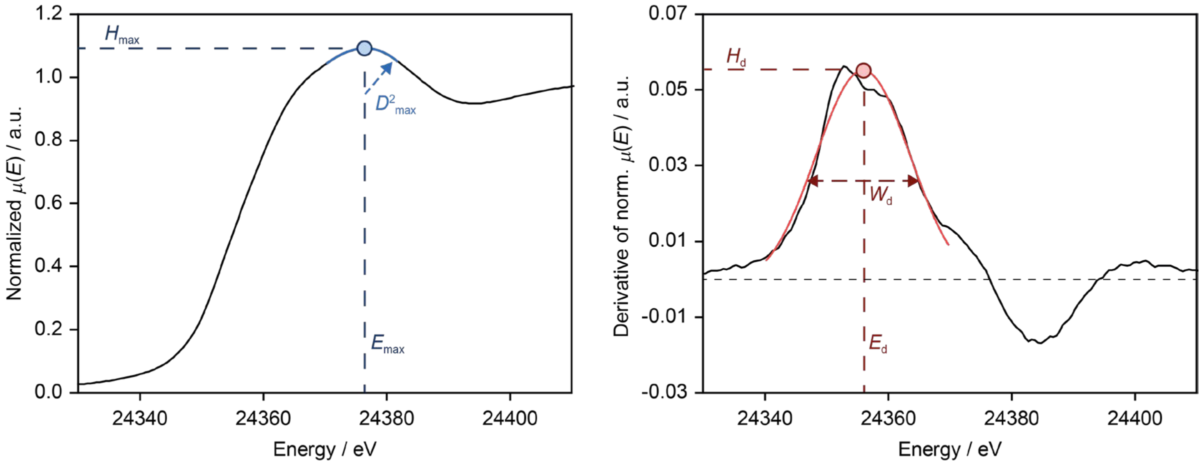


**Figure S2.** Graphical representation of the descriptors in **Table S1** exemplified using the in-situ signal of $Pd_1@C_3N_4$ in pure solvent (BH-1). Descriptors of the derivative spectra were obtained using a Gaussian fit of the first peak while a parabolic ft was used for XANES.

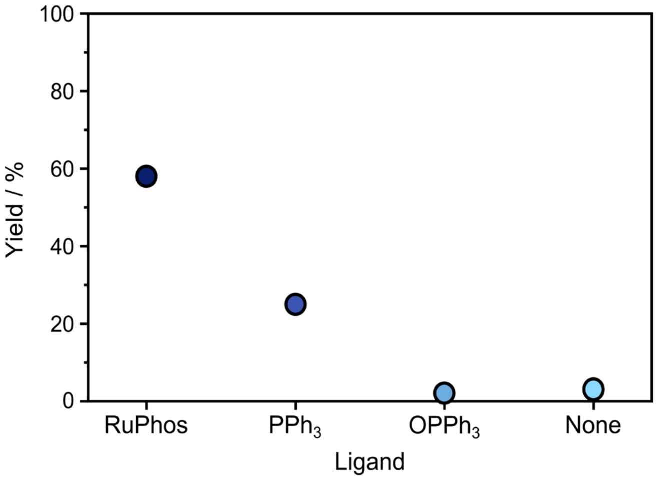


**Figure S3.** Comparison of ligand effect on reactivity using RuPhos, $PPh_3$, $OPPh_3$, and in the absence of ligand. Reaction conditions as described in **Table S2** using NaO$^t$Bu (3 equiv) as base and a duration of 20 h.

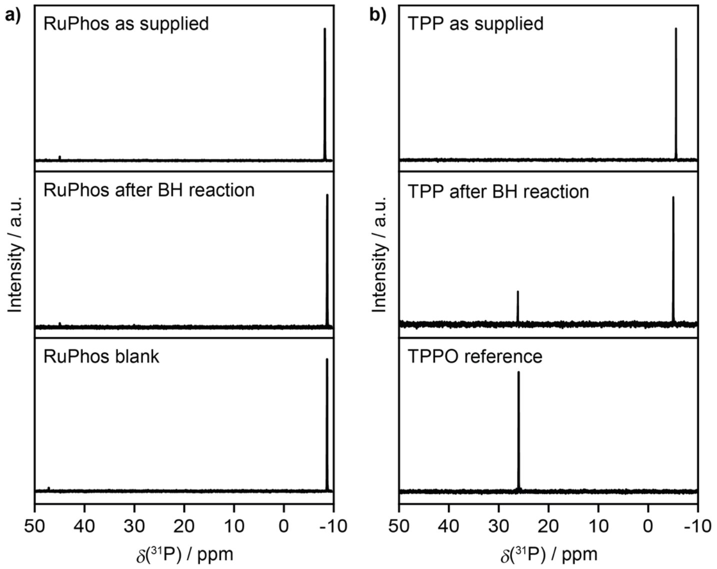


**Figure S4.** $^{31}$P NMR of **a)** RuPhos and **b)** $PPh_3$ ligands as supplied, used in the BH reaction, or used in a blank experiment (without catalyst), together with a $PPh_3O$ ligand as supplied for reference.

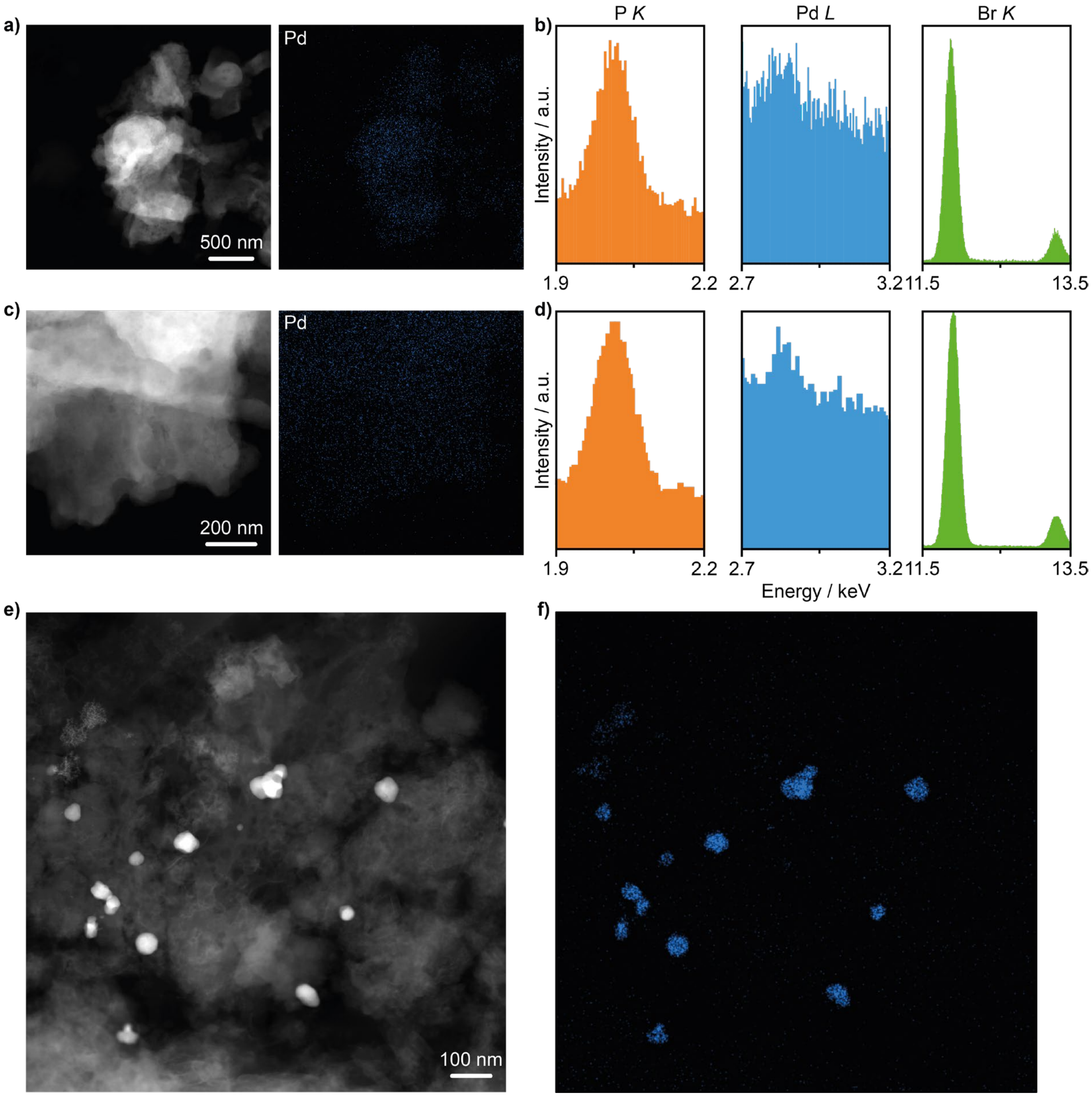


**Figure S5. a,c)** HAADF-STEM images of used $Pd_1@C_3N_4$ and corresponding elemental maps of Pd and **b,d)** corresponding energy dispersive X-ray spectra of P *K*-, Pd *L*-, and Br *K*-edge. Reaction conditions as described in **Table S2** using LiHMDS as base and RuPhos as ligand and a duration of 20 h, **e,f)** HAADF-STEM image and corresponding elemental map of $Pd_1@C_3N_4$ after exposure to conditions that promote nanoparticles formation (entry 7 in **Table S3**).

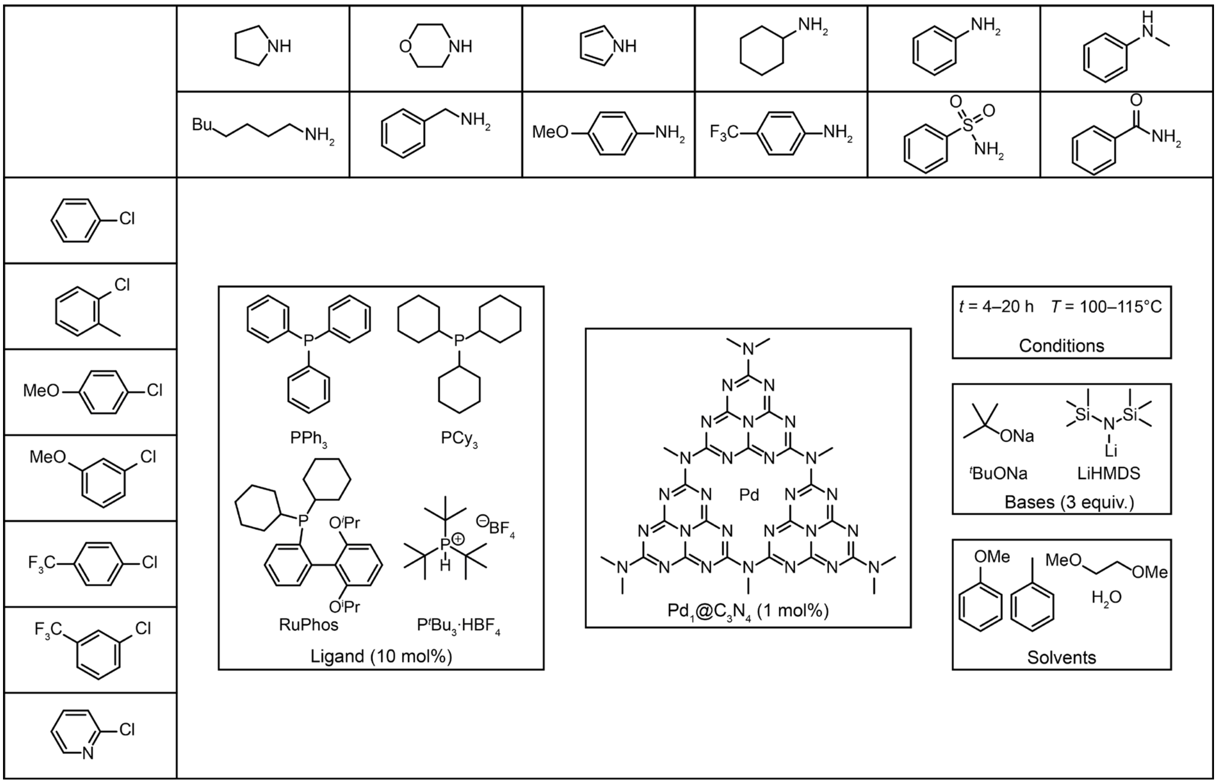


**Figure S6.** Schematic of the screening array performed following the standardized protocol for high throughput evaluation in BH coupling. Typical reaction conditions: Pd (0.01 equiv), ligand (0.1 equiv), base (3 equiv), Ar-X (top row, 1 equiv), amine (left column, 1.5 equiv), solvent (1 mL), 100-115°C, 4-20 h.

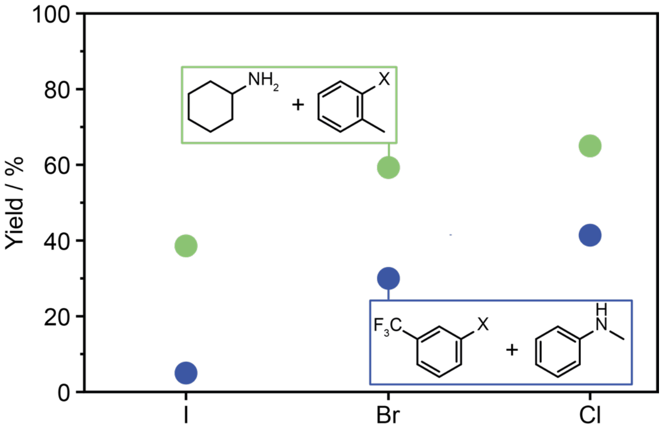


**Figure S7.** Comparative performance of $Pd_1@C_3N_4$ in the BH coupling of 2-halotoluenes with cyclohexylamine and 3-halo(trifluoromethyl)benzenes with *N*-methylamine. Reaction conditions as described in **Table S2** using NaO$^t$Bu as base and RuPhos as ligand and a duration of 4 h.

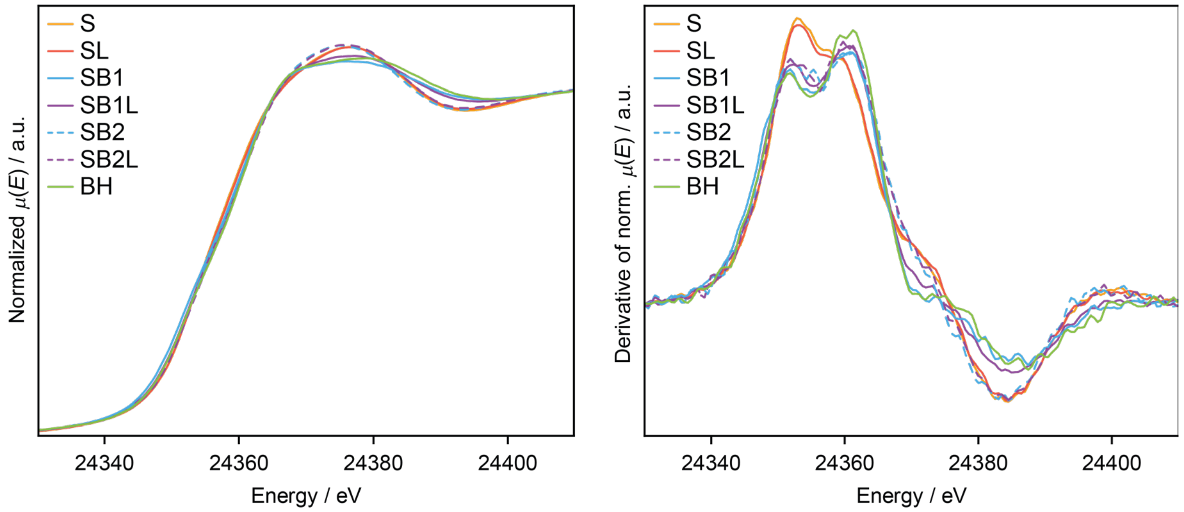


**Figure S8.** Derivative spectra (right) of the in-situ Pd *K*-edge XANES spectra also shown in **Figure 4a** (left**)**. Coding: solvent (S), base (B), ligand (L), and BH amination reaction (BH). Reaction conditions follow the standardized protocol shown in **Figure 2**, using RuPhos as a ligand, NaO$^t$Bu or LiHMDS as base, and morpholine and bromobenzene as reagents in the reaction.

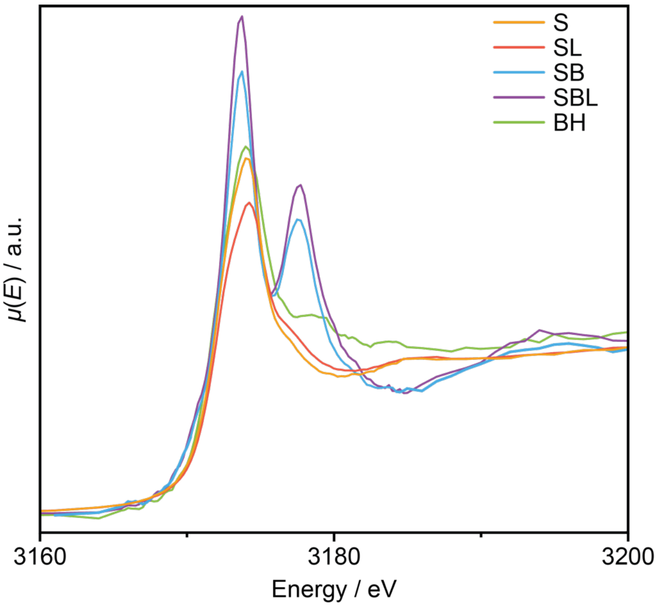


**Figure S9.** Ex-situ Pd $L_3$-edge XANES spectra of $Pd_1@C_3N_4$ treated in the presence of solvent (S), ligand (L), base (B) mixtures, and after the Buchwald-Hartwig reaction (BH) following the conditions described in **Table S2**. Catalysts were exposed to each mixture for 4 h before drop casting onto the sample holder.

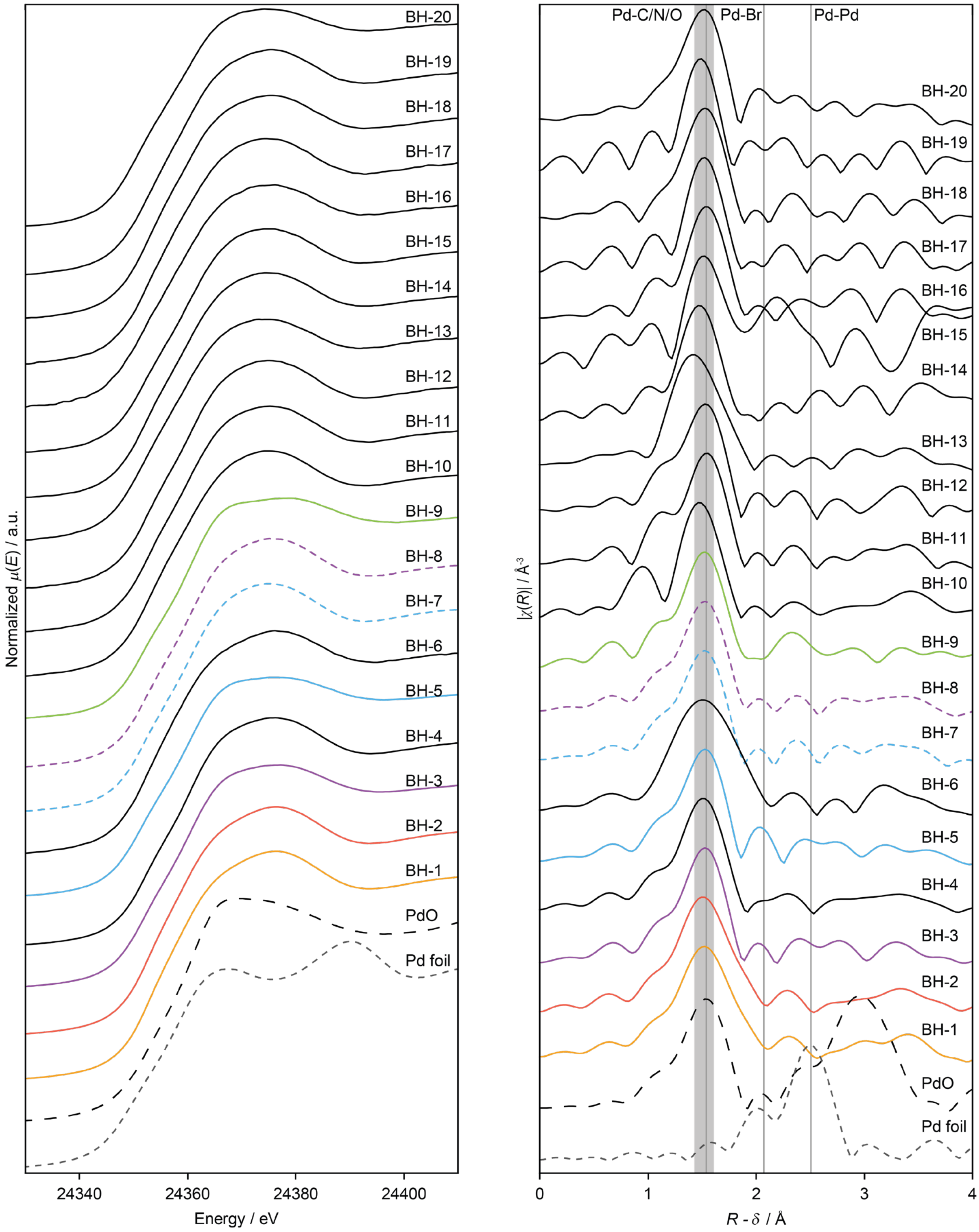


**Figure S10.** In-situ Pd *K*-edge XANES and corresponding EXAFS spectra of the $Pd_1@C_3N_4$ catalyst in all tested conditions according to **Table S1**. The spectra shown in the main manuscript are shown with the same color code while the others are left black. References are dashed. The grey area and grey dashed line indicate the relevant scattering paths.

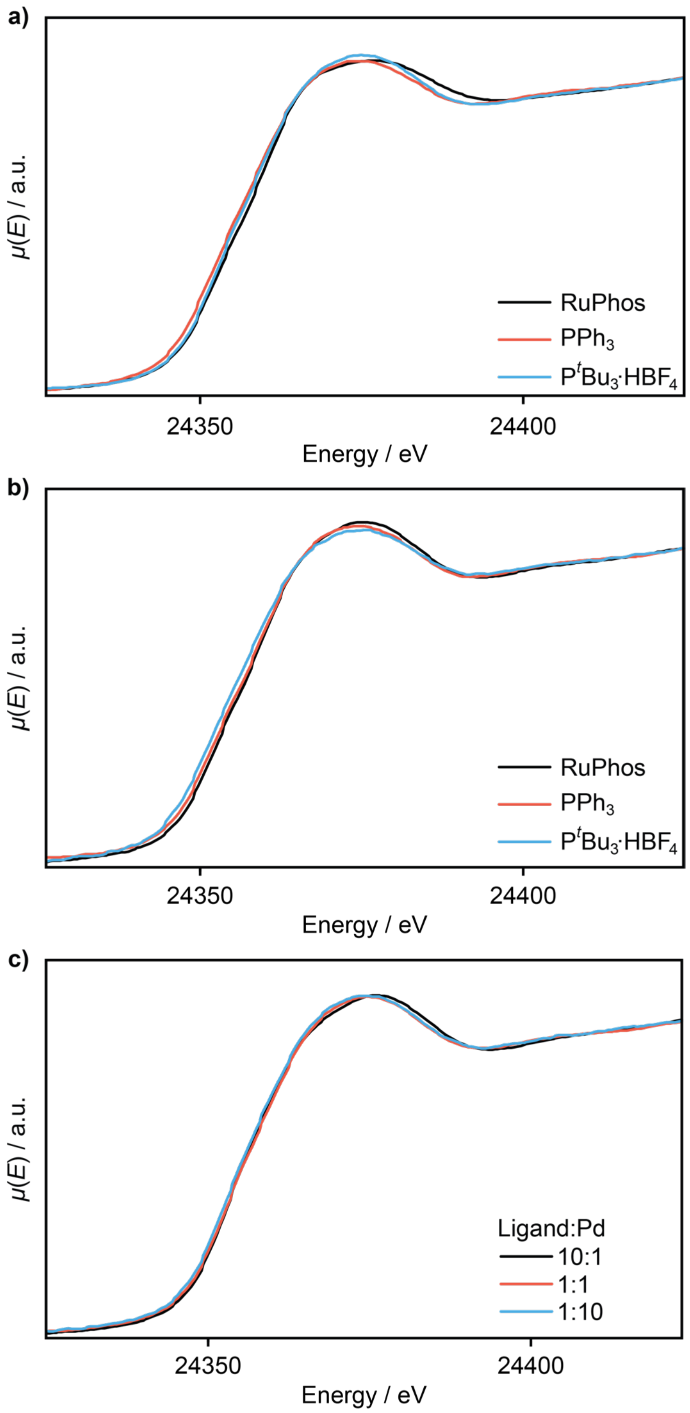


**Figure S11.** In-situ Pd *K*-edge XANES spectra of the $Pd_1@C_3N_4$ catalyst in the BH reaction using distinct ligands with **a)** LiHMDS or **b)** NaO$^t$Bu as the base. **c)** Varying the ligand:Pd ratio, using RuPhos as ligand and NaO$^t$Bu as the base. Other reaction conditions as described in **Table S2** with a duration of 4 h.

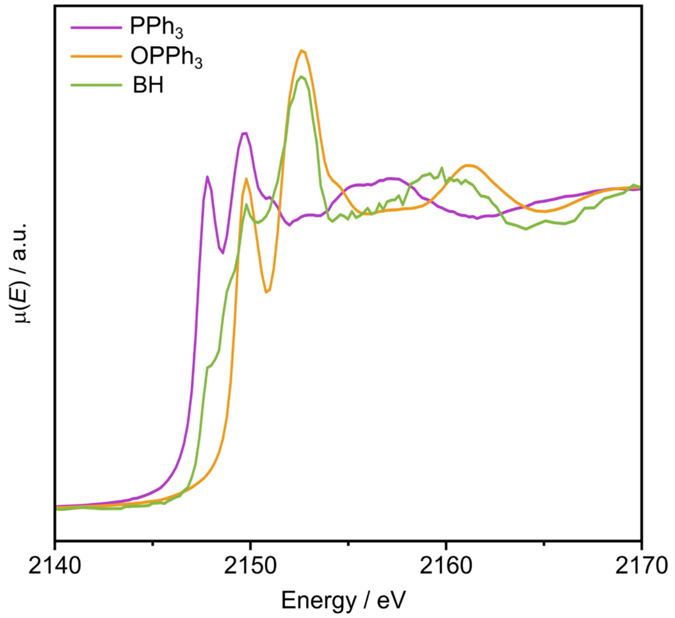


**Figure S12.** Ex-situ P *K*-edge XANES spectra of $Pd_1@C_3N_4$ after use in the BH coupling of bromobenzene and morpholine with LIHDMS base and $PPh_3$ ligand following the procedure described in **Table S2** for 4 h. Triphenylphosphine ($PPh_3$) and triphenylphosphine oxide ($OPPh_3$) are used as references.

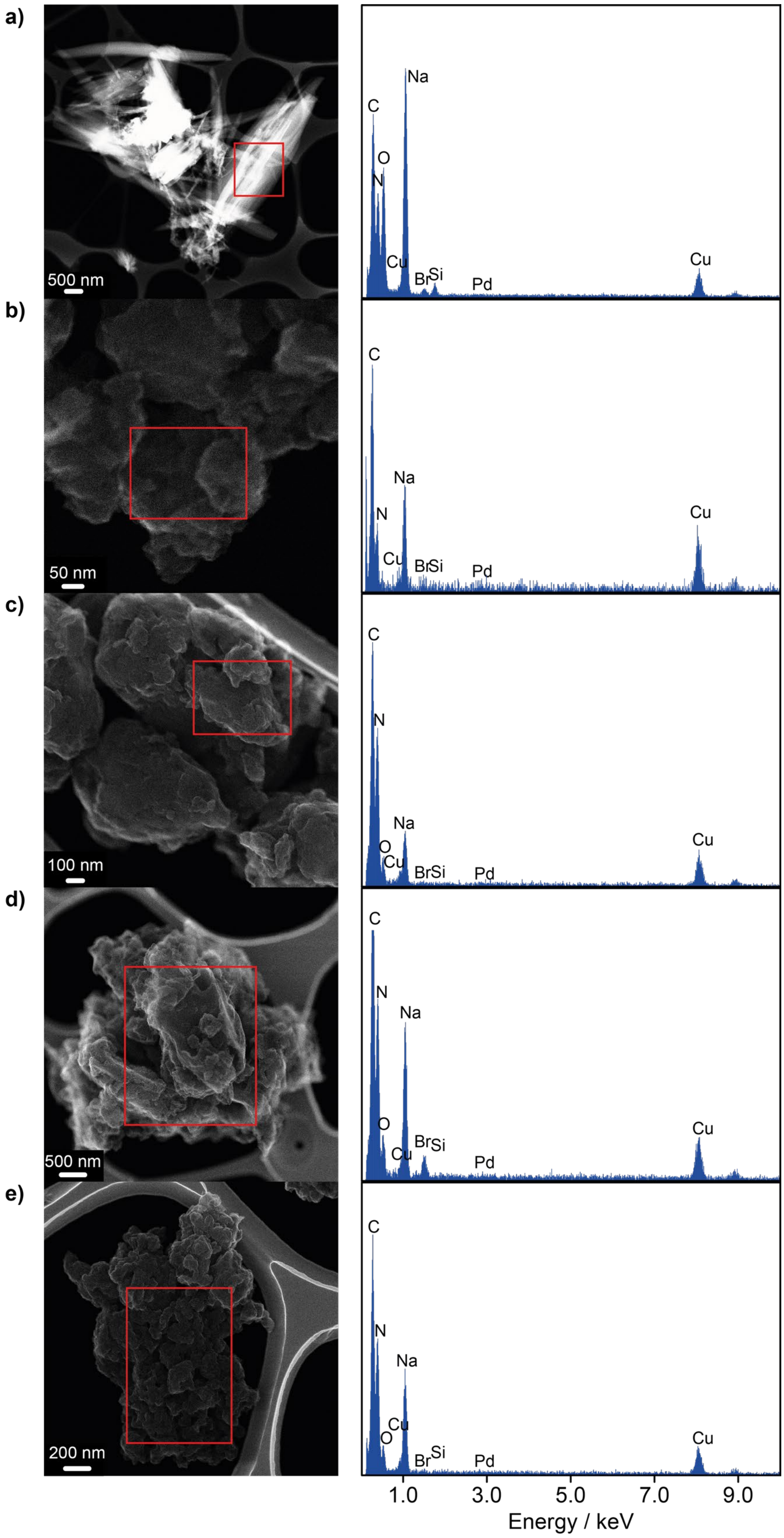


**Figure S13.** Low-magnification HAADF-STEM images (left) and corresponding EDX spectra (right) of **a)** the used $Pd_1@C_3N_4$ catalyst recovered after use in the BH reaction of morpholine with bromobenzene, and **b-e)** the used catalyst after washing with ethanol **(b)**, water **(c)**, acetonitrile **(d)**, or methanol **(e)**.

**Supplementary Data 1: QNMR Spectra of Products**

**Note S2 | Comment on NMR Spectra.** The following spectra are quantitative NMR measurements of the reaction mixture. 1,3,5-trimethoxybenzne was added as an internal standard. The singlet methoxy peak at $\delta = 3.34$ was used for quantification by setting its area to 9 and is reported for every spectrum. Furthermore, a peak belonging to the product is always reported. Its integral does not correspond to the number of hydrogen atoms in the molecule but instead the abundance compared to the standard.

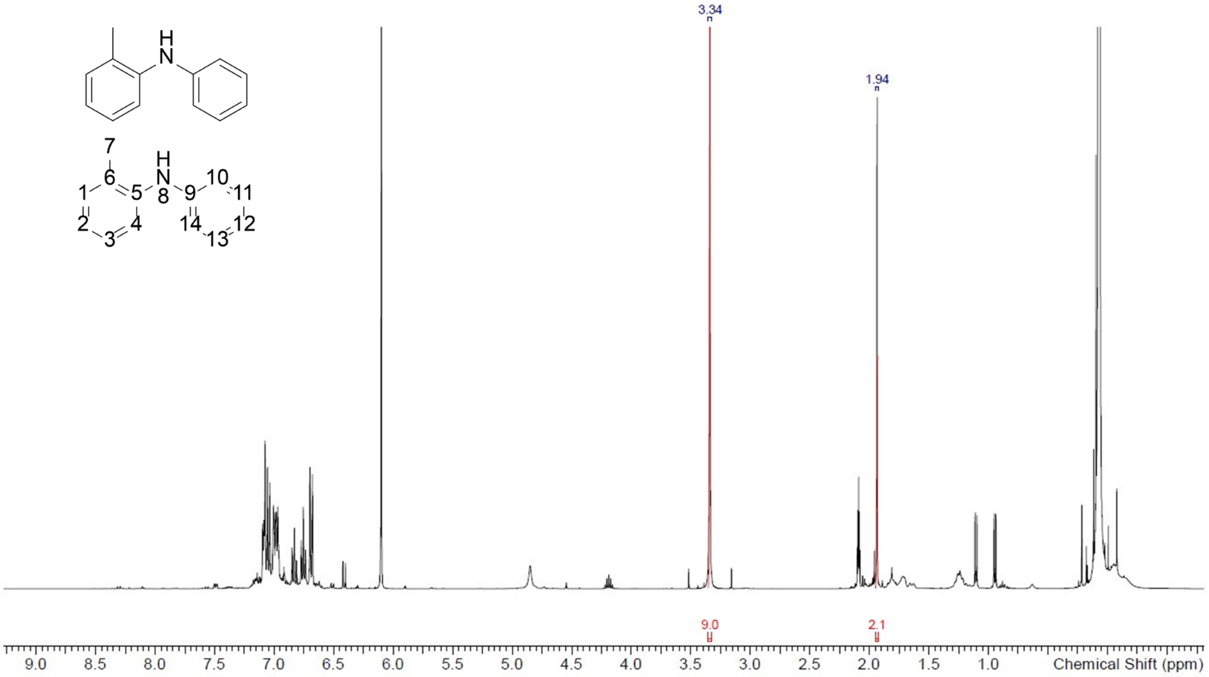


$^{1}$H NMR (400 MHz, toluene) spectrum of 2-methyl-N-phenylaniline $\delta = 3.34$ (s, 9H), 1.94 (s, 2H). The compound was synthesized by standardized protocol A from 1-iodo-2-methylbenzene and aniline.

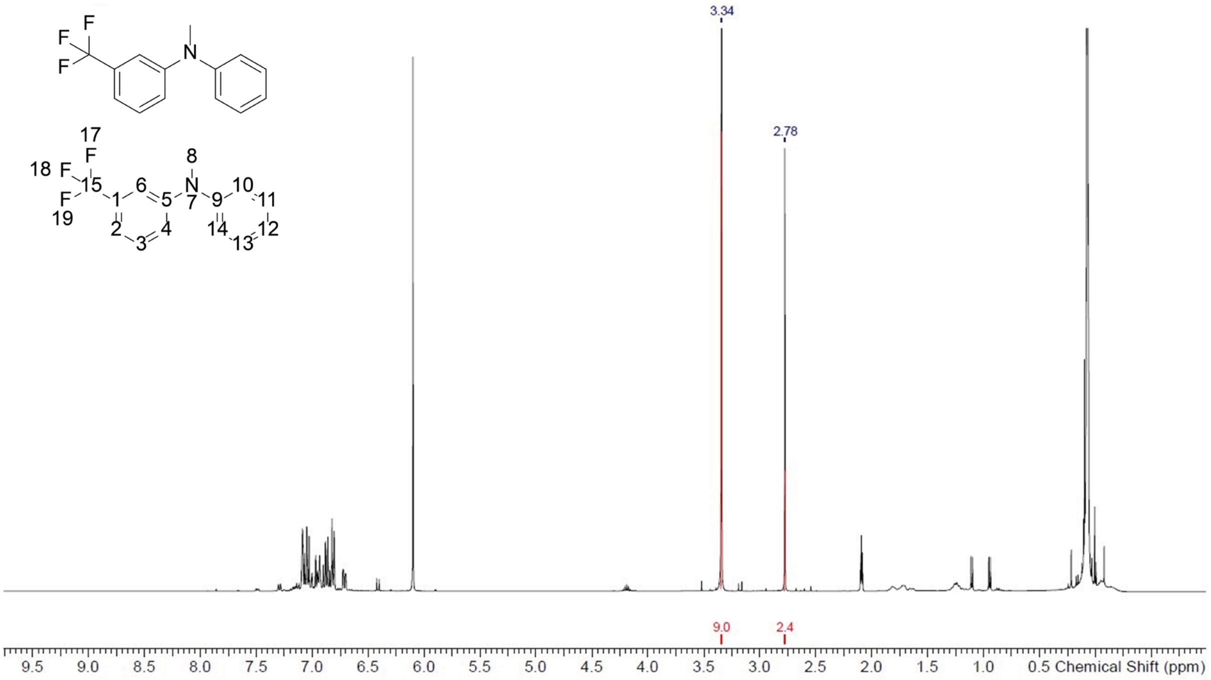


$^{1}$H NMR (400 MHz, toluene) spectrum of N-methyl-N-[3-(trifluoromethyl)phenyl]amine $\delta = 3.34$ (s, 9H), 2.78 (s, 2H). The compound was synthesized by standardized protocol A from 1-iodo-3-(trifluoromethyl)benzene and N-methylaniline.

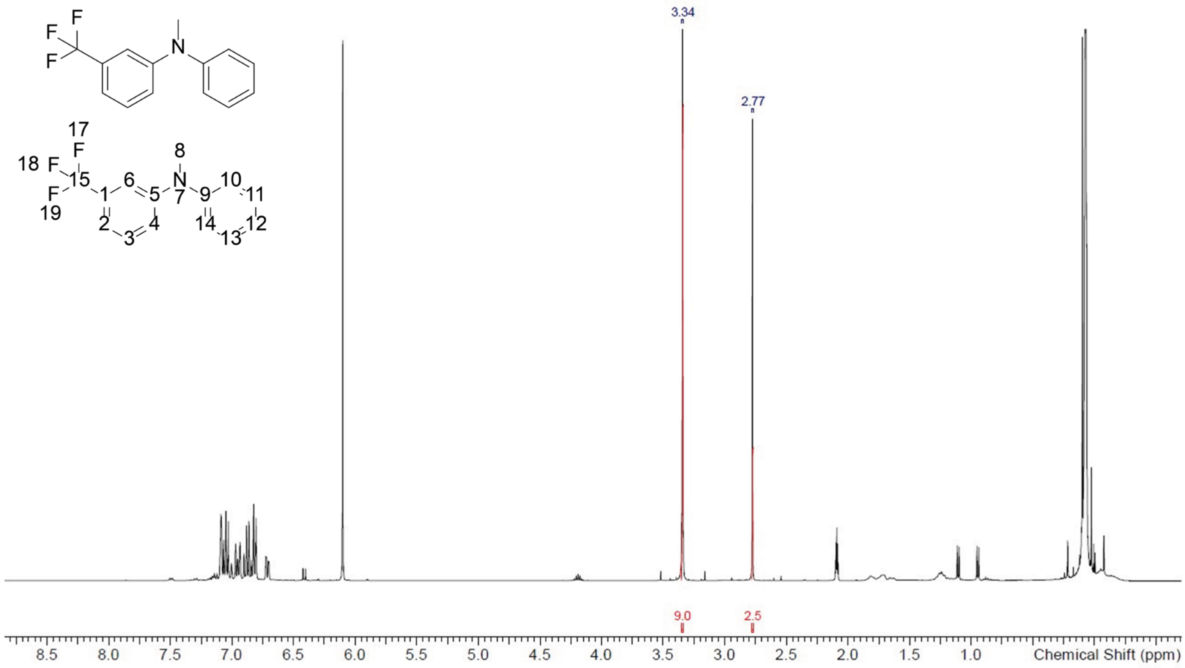


$^{1}$H NMR (400 MHz, toluene) spectrum of N-methyl-N-[3-(trifluoromethyl)phenyl]amine $\delta = 3.34$ (s, 9H), 2.77 (s, 2H). The compound was synthesized by standardized protocol A from 1-chloro-3-(trifluoromethyl)benzene and N-methylaniline.

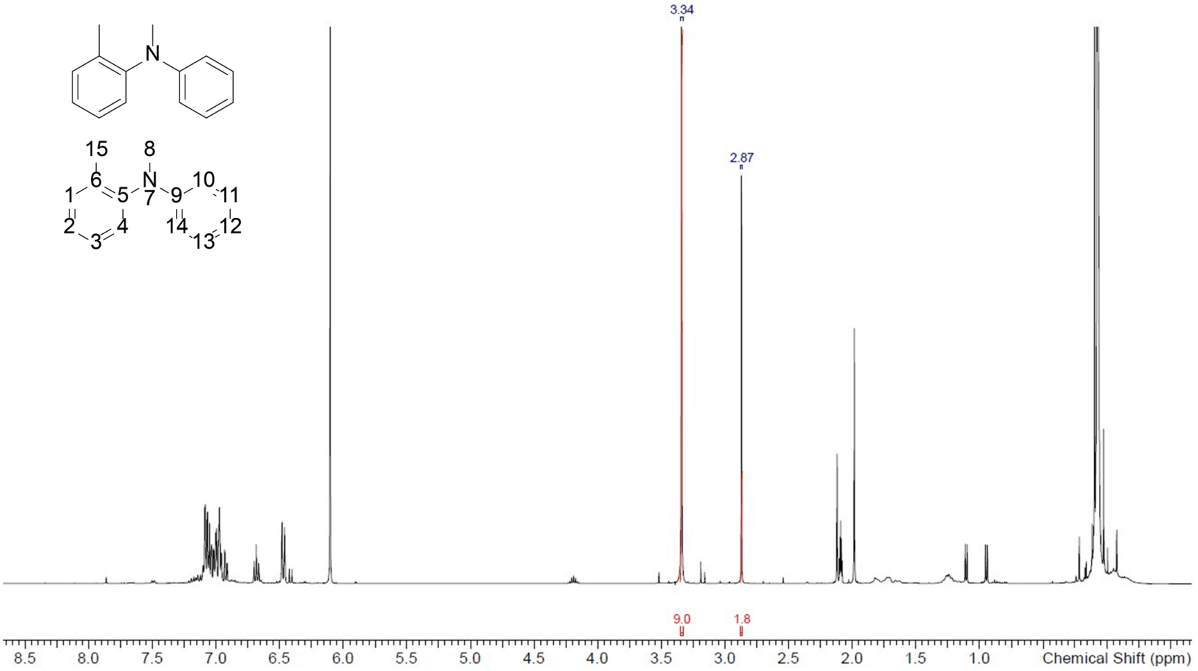


$^{1}$H NMR (400 MHz, toluene) spectrum of N,2-dimethyl-N-phenylaniline $\delta = 3.34$ (s, 9H), 2.87 (s, 2H). The compound was synthesized by standardized protocol A from 1-chloro-2-methylbenzene and N-methylaniline.

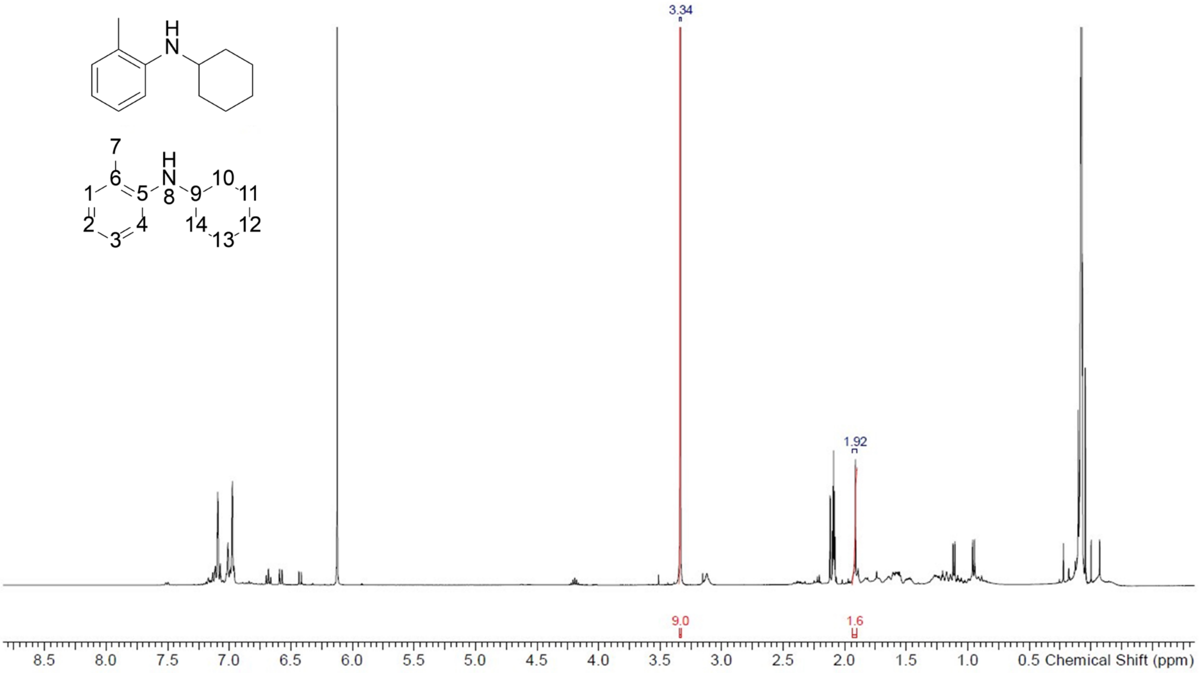


$^{1}$H NMR (400 MHz, toluene) spectrum of N-cyclohexyl-3-methylaniline $\delta = 3.34$ (s, 9H), 1.92 (s, 2H). The compound was synthesized by standardized protocol A from 1-bromo-2-methylbenzene and cyclohexylamine.

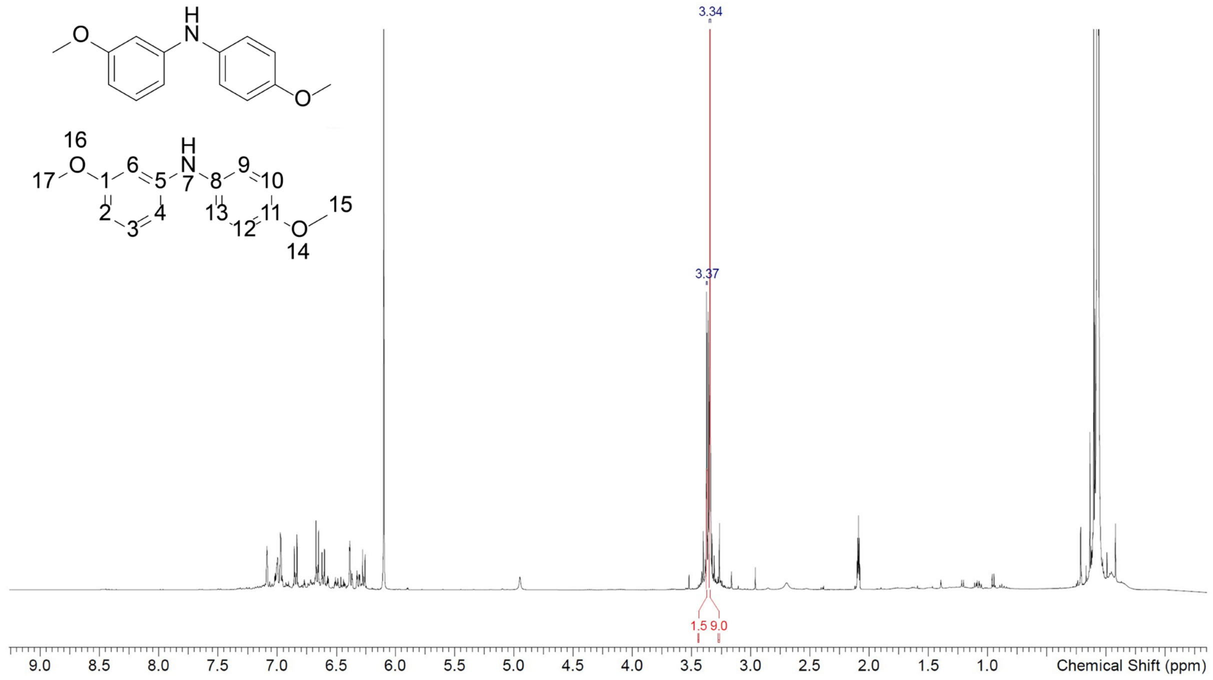


$^{1}$H NMR (400 MHz, toluene) spectrum of 3,4'-dimethoxydiphenylamine $\delta$= 3.37 (s, 2H), 3.34 (s, 9H). The compound was synthesized by standardized protocol A from 3-iodo-anisole and 4-methoxyaniline.

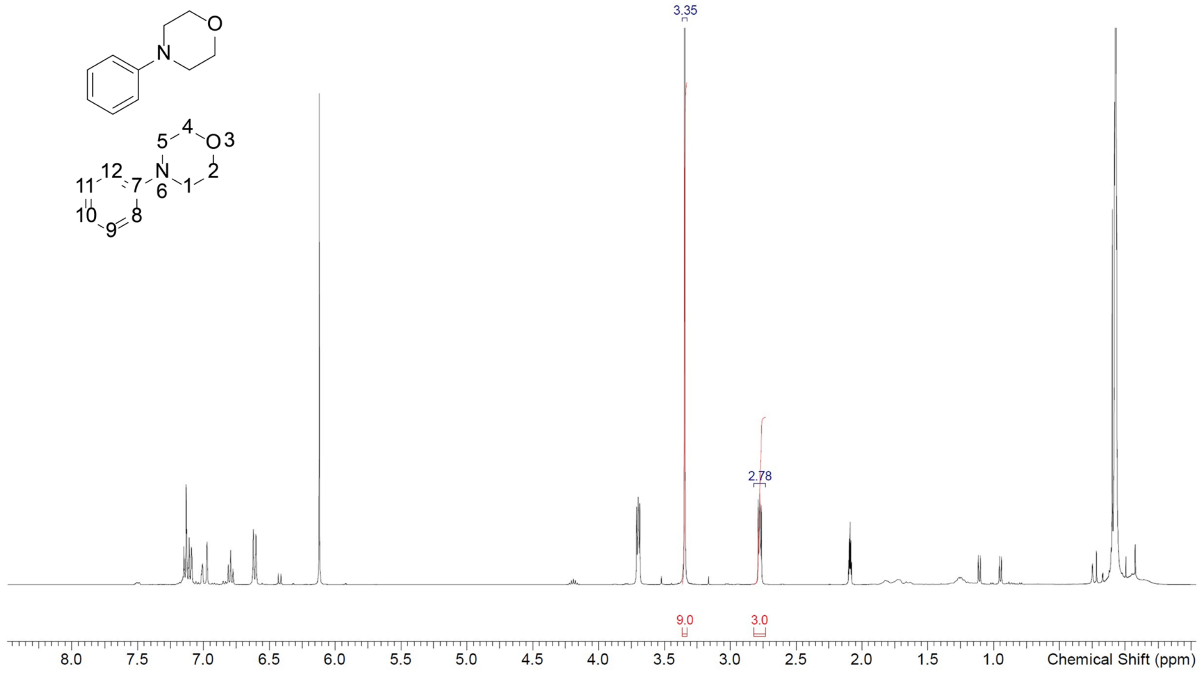


$^{1}$H NMR (400 MHz, toluene) spectrum of 4-phenylmorpholine $\delta$= 3.35 (s, 9H), 2.82-2.73 (m, 3H). The compound was synthesized by standardized protocol A from iodobenzene and morpholine.

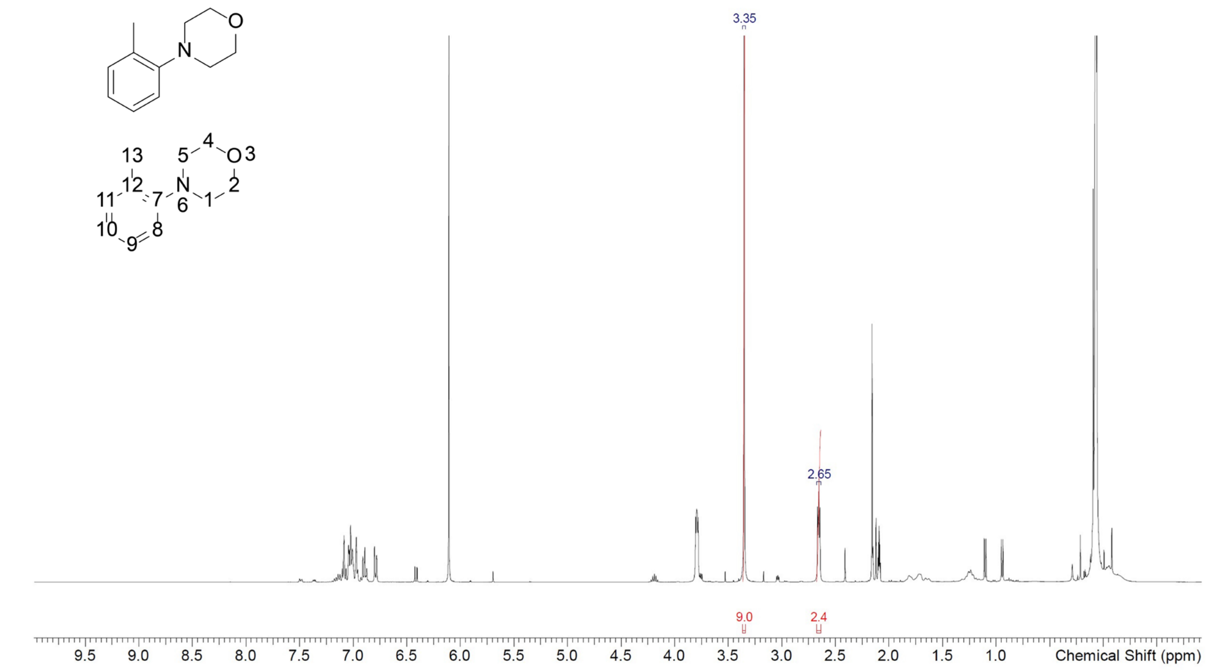


$^{1}$H NMR (400 MHz, toluene) spectrum of 4-phenylmorpholine $\delta = 3.35$ (s, 9H), 2.68-2.63 (m, 2H). The compound was synthesized by standardized protocol A from 1-iodo-2-methylbenzene and morpholine.

## References


(1) Veisi, H.; Tamoradi, T.; Karmakar, B.; Hemmati, S. Green Tea Extract–Modified Silica Gel Decorated with Palladium Nanoparticles as a Heterogeneous and Recyclable Nanocatalyst for Buchwald-Hartwig C–N Cross-Coupling Reactions. *J. Phys. Chem. Solids* **2020**, *138*, 109256. DOI:10.1016/j.jpcs.2019.109256

(2) Sarvestani, M.; Azadi, R. Buchwald-Hartwig Amination Reaction of Aryl Halides using Heterogeneous Catalyst Based on Pd Nanoparticles Decorated on Chitosan Functionalized Graphene Oxide *Appl. Organomet. Chem*. **2018**, 32, 1–9. DOI:10.1002/aoc.3906

(3) Panahi, F.; Daneshgar, F.; Haghighi, F.; Khalafi-Nezhad, A. T Immobilized Pd Nanoparticles on Silica-Starch Substrate (PNP-SSS): Efficient Heterogeneous Catalyst in Buchwald–Hartwig C–N Cross Coupling Reaction. *J. Organomet. Chem.* **2017**, *851*, 210–217. DOI:10.1016/j.jorganchem.2017.09.037

(4) Chen, Z.; Wang, S.; Lian, C.; Liu, Y.; Wang, D.; Chen, C.; Peng, Q.; Li, Y. Nano PdAu Bimetallic Alloy as an Effective Catalyst for the Buchwald–Hartwig Reaction. *Chem. Asian J.* **2016**, *11*, 351–355. DOI:10.1002/asia.201500531

(5) Fareghi-Alamdari, R.; Haqiqi, M. G.; Zekri, N. Immobilized Pd(0) Nanoparticles on Phosphine-Functionalized Graphene as a Highly Active Catalyst for Heck, Suzuki and N-Arylation Reactions. *New J. Chem.* **2016**, 40, 1287–1296. DOI: 10.1039/C5NJ02227D

(6) Woo, S.; Kim, Y.; Woo, S.; Kim, M.; Kim, Y.; Hong, W.; Ahn, S. The Facet-Dependent Enhanced Catalytic Activity of Pd Nanocrystals. *Chem. Commun.* **2014**, *50*, 9454–9457. DOI:10.1039/C4CC02494J

(7) Xiao, Q.; Sarina, S.; Bo, A.; Jia, J.; Liu, H.; Arnold, D. P.; Huang, Y.; Wu, H.; Zhu, H. Visible Light-Driven Cross-Coupling Reactions at Lower Temperatures Using a Photocatalyst of Palladium and Gold Alloy Nanoparticles. *ACS Catal.* **2014**, *4*, 1725–1734. DOI: 10.1021/cs5000284

(8) Prabhu, R. N.; Ramesh, R. Synthesis and Structural Characterization of Palladium(II) Thiosemicarbazone Complex: Application to the Buchwald–Hartwig Amination Reaction. *Tetrahedron Lett.* **2013**, *54*, 1120–1124. DOI:10.1016/j.tetlet.2012.12.070

(9) Maiti, D.; Fors, B.; Henderson, P. J. L.; Nakamura, Y.; Buchwald, S. L. Palladium-Catalyzed Coupling of Functionalized Primary and Secondary Amines with Aryl and Heteroaryl Halides: Two Ligands Suffice in Most Cases. *Chem. Sci.* **2011**, *2*, 57–68. DOI:10.1039/C0SC00330A

(10) Majumder, A.; Gupta, R.; Mandal, M.; Babu, M. Air-Stable Palladium(0) Phosphine Sulfide Catalysts for Ullmann-Type C–N and C–O Coupling Reactions. *J. Organomet. Chem.* **2015**, *781*, 23–34. DOI:10.1016/j.jorganchem.2014.11.018

(11) Dai, Q.; Gao, W.; Liu, D.; Kapes, L. M.; Zhang, X. Triazole-Based Monophosphine Ligands for Palladium-Catalyzed Cross-Coupling Reactions of Aryl Chlorides. *J. Org. Chem.* **2006**, *71*, 3928–3934. DOI:10.1021/jo060321e

(12) Osorio-Tejada, J. L.; Ferlin, F.; Vaccaro, L.; Hessel, V. The Sustainability Impact of Nobel Prize Chemistry: Life Cycle Assessment of C–C Cross-Coupling Reactions. *Green Chem.* **2023**, *25*, 9760–9778. DOI:10.1039/D3GC01896B.
